# Bacterial Nucleotidyl Cyclases Activated by Calmodulin or Actin in Host Cells: Enzyme Specificities and Cytotoxicity Mechanisms Identified to Date

**DOI:** 10.3390/ijms23126743

**Published:** 2022-06-16

**Authors:** Magda Teixeira-Nunes, Pascal Retailleau, Martine Comisso, Vincent Deruelle, Undine Mechold, Louis Renault

**Affiliations:** 1 Institute for Integrative Biology of the Cell (I2BC), CEA, CNRS, Université Paris-Saclay, 91198 Gif-sur-Yvette, France; magda.teixeira-nunes@i2bc.paris-saclay.fr (M.T.-N.); martine.comisso@i2bc.paris-saclay.fr (M.C.); 2 Institut de Chimie des Substances Naturelles (ICSN), CNRS-UPR2301, Université Paris-Saclay, 1 Avenue de la Terrasse, 91198 Gif-sur-Yvette, France; pascal.retailleau@cnrs.fr; 3 Unité de Biochimie des Interactions Macromoléculaires, Département de Biologie Structurale et Chimie, CNRS UMR 3528, Institut Pasteur, 75015 Paris, France; vincent.deruelle@pasteur.fr (V.D.); undine.mechold@pasteur.fr (U.M.)

**Keywords:** bacterial nucleotidyl cyclase toxins, *Pseudomonas aeruginosa* Exoenzyme Y (ExoY), *Bacillus anthracis* Edema Factor (EF), *Bordetella pertussis* CyaA, cAMP-dependent signaling, actin cystoskeleton, cytoskeletoxins, host–pathogen interactions

## Abstract

Many pathogens manipulate host cell cAMP signaling pathways to promote their survival and proliferation. Bacterial Exoenzyme Y (ExoY) toxins belong to a family of invasive, structurally-related bacterial nucleotidyl cyclases (NC). Inactive in bacteria, they use proteins that are uniquely and abundantly present in eukaryotic cells to become potent, unregulated NC enzymes in host cells. Other well-known members of the family include *Bacillus anthracis* Edema Factor (EF) and *Bordetella pertussis* CyaA. Once bound to their eukaryotic protein cofactor, they can catalyze supra-physiological levels of various cyclic nucleotide monophosphates in infected cells. Originally identified in *Pseudomonas aeruginosa*, ExoY-related NC toxins appear now to be more widely distributed among various γ- and β-proteobacteria. ExoY-like toxins represent atypical, poorly characterized members within the NC toxin family. While the NC catalytic domains of EF and CyaA toxins use both calmodulin as cofactor, their counterparts in ExoY-like members from pathogens of the genus *Pseudomonas* or *Vibrio* use actin as a potent cofactor, in either its monomeric or polymerized form. This is an original subversion of actin for cytoskeleton-targeting toxins. Here, we review recent advances on the different members of the NC toxin family to highlight their common and distinct functional characteristics at the molecular, cytotoxic and enzymatic levels, and important aspects that need further characterizations.

## 1. Introduction

**Figure 1:**
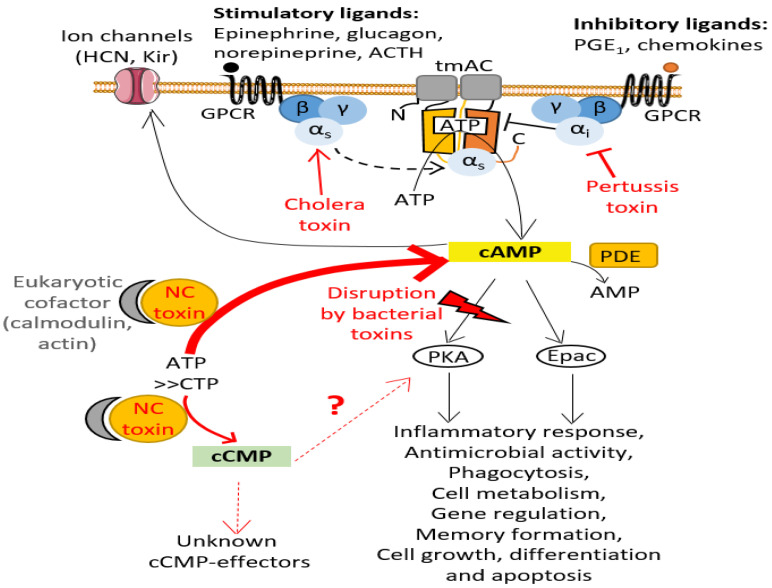
cAMP signaling pathway and its disruption by bacterial toxins. cAMP is synthesized by transmembrane AC (tmAC) (soluble AC, sAC, is not represented here), which are activated by the binding of an agonist to a G protein-coupled receptors (GPCRs) stimulating the transition of the heterotrimeric G protein alpha subunits Gαs into its active GTP-bound state (see text for more details). cAMP activates various effectors such as protein kinase A (PKA) and guanine nucleotide-exchange Epac, which then regulate various cellular processes, including immune system responses. cAMP can also activate ion channels. Phosphodiesterases (PDE) regulate the degradation of cAMP by catalyzing its conversion into AMP. Bacterial pathogens have developed several strategies to manipulate the cAMP levels within host cells to disrupt intracellular signaling [1]: they can inject toxins (in red) into host cells that either alter the Gα subunits responsible for activating or inhibiting eukaryotic tmAC, thus preventing host cells to properly regulate tmAC enzymatic activity (e.g., pertussis toxin (PT), cholera toxin (CT)), or directly produce uncontrolled, toxic levels of cAMP or other cNMPs (such as cCMP) once these NC toxins are specifically activated by their eukaryotic cofactor.

The canonical cyclic nucleotides cAMP and cGMP are universal intracellular second messengers that relay external signals from cell-surface receptors to effector proteins within eukaryotic or bacterial cells. These 3′,5′-cyclic monophosphate nucleotides (cNMPs) can locally activate one or more effectors to trigger complex signaling cascades in cells that involve amplifications of chemical signals, intertwined and highly cell type-dependent signaling networks. cNMP synthesis, cNMP levels and cNMP degradation must be precisely controlled in cells, both temporally and spatially, to enable precise regulation of cell signaling. Many pathogens manipulate the cAMP levels of host cells for their own benefit (see [1] for a thorough overview by bacterial, fungal and protozoan pathogens). To successfully subvert host cell signaling, bacterial pathogens have developed multiple strategies [1]. They can take control of the activity of eukaryotic adenylate cyclases (ACs), the nucleotidyl cyclase (NC) enzymes that catalyze the conversion of ATP into Adenosine 3′,5′-cyclic monophosphate (cAMP) and pyrophosphate (PPi), by acting on diverse levels of signal transduction in the host cell [1,2]. One of their strategies is to inject exotoxins into eukaryotic host cells that prevent physiological regulation of endogenous host AC activity. In eukaryotic cells, the enzymatic activity of ubiquitous plasma membrane-associated AC (transmembrane AC or tmAC) is stimulated or inhibited by the active (GTP-bound) conformation of stimulatory (Gαs) or inhibitory (Gαi) Gα subunits of heterotrimeric G proteins, respectively (Figure 1). The cholera toxin (CT) from *Vibrio cholerae* and pertussis toxin (PT) from *Bordetella pertussis* correspond to oligomeric exotoxins containing an ADP-ribosylating subunit (A protomer). Inside the host cell, the latter covalently transfers an ADP-ribose moiety from NAD+ (nicotinamide adenine dinucleotide) to either a Gαs arginine (Arg201 in Gsα2 or Arg147 in Gtα) or Gαi cysteine (Cys351 in Gi1-i3α) residue, respectively [2,3]. Consequently, CT ADP-ribosylation modification creates constitutively-active Gαs subfamily that up-regulates host plasma membrane-associated AC activity, whereas PT modification locks Gαi subfamily into a constitutively-inactive state that cannot down-regulate AC activity (Figure 1). The biological effects resulting from this dysregulation of eukaryotic plasma membrane-associated AC activity vary widely among cell types [2,3]. For example, the ADP-ribosylation activity of PT has been shown to inhibit neutrophil transendothelial migration and skin vascular permeability in vivo in a rat model of inflammation [4].

Another common strategy developed by bacterial pathogens to generate uncontrolled toxic levels of cAMP within host cells is the injection or translocation of bacterial adenylate cyclases into eukaryotic cells [1,5,6,7]. The archetype members of this family of secreted invasive bacterial NC were identified in the 1980s in *Bacillus anthracis* and *Bordetella pertussis* bacteria, which cause anthrax and whooping cough in humans, and secrete Edema Factor (EF) [8] and CyaA [9] AC exotoxins, respectively. The family also includes ExoY-like NC toxins. Its founding member, exotoxin Y (ExoY) and its intrinsic AC activity were identified nearly 20 years later in *Pseudomonas aeruginosa* (*P. a.*) based on shared sequence features with the AC domains of EF and CyaA [10]. *P. aeruginosa* represents a major opportunistic and nosocomial human pathogen, listed by the World Health Organization (WHO) as being among the three most critical multidrug-resistant pathogens. *P. aeruginosa* ExoY toxin (hereinafter referred to as *Pa*-ExoY) is among the effectors injected by the type 3 secretion system (T3SS) into eukaryotic cells. The high prevalence of the *exoY* gene among genomes of largely diverse *P. aeruginosa* strains [11,12,13,14] suggests an important role of ExoY in pathogenicity of *P. aeruginosa*. More recently, secreted ExoY-related effector domains or proteins have also been found among the virulence factor arsenal of various Gram-negative proteobacteria [15,16,17]. EF, CyaA and ExoY-like exotoxins have structurally related catalytic AC domains, which are non-homologous to the hetero- or homo-dimeric catalytic domains of eukaryotic AC defining the class III of AC [6,18]. Based on their common sequence features, these bacterial AC enzymes define the class II of AC that includes only AC secreted by Gram-positive (EF) or Gram-negative (CyaA, ExoY-like) bacteria [18]. Other pathogens express bacterial AC toxins to facilitate their survival in hosts, such as *Yersinia pestis* the causative agent of plague in humans [1,6,18]. However, the AC toxin of *Y. pestis* is structurally unrelated to bacterial class II AC, but belongs to AC class IV described elsewhere [5,6,18,19], and remains inside the pathogen during infection [20].

ExoY-like NC toxins represent less-studied and atypical members of the class II family of AC toxins. They use a cofactor that is original in this family, namely monomeric or polymerized actin [17,21]. Actin is a frequent target of bacterial and viral effector proteins, which can affect the polymerization state of actin in different ways to the advantage of the pathogen by introducing chemical modifications on actin, such as ADP-ribosylation [22] or crosslinking [23]. Its use as a cofactor that strongly stimulates the catalytic activity of the NC enzyme represents an original way to target actin among cytoskeletoxins, i.e., effector proteins of pathogens that target the host cell cytoskeleton. To our knowledge, the only known examples of bacterial effectors that use actin as a cofactor are ExoY-like NC toxins and the *Yersinia* outer protein YopO/YpkA [24]. Here, we review the main functional and enzymatic features and the mechanisms of cytotoxicity and virulence known to date for representative members of the secreted class II AC family, i.e., the canonical EF and CyaA and the atypical ExoY-like NC toxins. We highlight important aspects that need further characterizations for all members or for actin-activated ExoY-like NC toxins. The functional and virulence features of ExoY NC toxins in various bacterial infections are still largely unknown and need to be better defined and we aim to provide a baseline with our review.

## 2. The Key Enzymatic Properties That Allow Bacterial Class II Adenylate Cyclases to Be Highly Subversive to Host Cells

In the course of the evolutionary selection of class II bacterial secreted AC toxins, pathogens appear to have optimized and combined at least three common enzymatic features.

**Figure 2:**
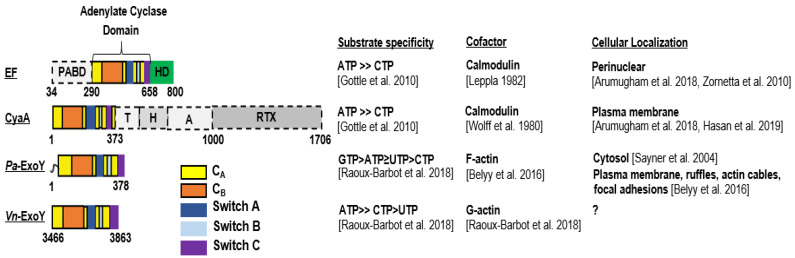
Domain organization, substrate specificity, activation cofactor and cellular localization of CaM-activated toxins CyaA and EF and actin-activated toxins, *Pa*-ExoY and *Vn*-ExoY. PABD, protective antigen binding domain; HD, helical domain; T, translocation region; H, hydrophobic region; A, acylation region; RTX, repeat-in-toxin [37,38,39]. (Note: ATP ≥ CTP [34]; GTP > ATP ≥ UTP > CTP and ATP ≥ CTP > UTP [21]; Calmodulin [8,9]; F-actin [17]; G-actin [21]; Perinuclear [40,41]; Plasma membrane [40,42]; Cytosol [43]; Plasma membrane, ruf-fles, actin cables, focal adhesions [17]).

First, since the nucleotide triphosphate substrates of these toxins are not unique to eukaryotic organisms, it is essential for bacteria to ensure that the bacterial effectors are kept inactive inside bacterial cells and become active only in infected eukaryotic cells. Class II AC toxins, as isolated enzymes, exhibit a very low basal catalytic activity. Once toxins have entered the eukaryotic host cell, their enzymatic activity is strongly stimulated by contact with a host cell cofactor, which is usually a specific, ubiquitous, highly conserved protein of the host. Provided that the eukaryotic cofactor is present in the delivery compartment of the bacterial effector at a suitable concentration for the interaction to occur, the bacterial AC can be constitutively activated and be harmful for the host. EF and CyaA class II AC share the same cofactor, namely the Ca^2+^-sensing protein calmodulin [8,9]. This small eukaryotic protein of 16.7 kDa is involved in calcium-mediated signal networks, highly conserved through evolution and abundant in the cytoplasm of all higher cells, with a total cellular concentration ranging from 2 to 25 μM depending on mammalian tissues [25]. It so happens that mammalian structurally-unrelated transmembrane ACs (AC1, AC8) are also regulated by Ca^2+^ and calmodulin [26]. In contrast, (some) ExoY-like class II AC use an original cofactor that was only recently identified as the eukaryotic 42-kDa protein actin in its filamentous (F-actin) or monomeric (G-actin) state [17,21] (Figure 2). Actin perfectly meets the criteria for a cofactor that triggers cytotoxic enzyme activity only in host cells: it is absent in bacteria, very well conserved, one of the most abundant proteins and widely distributed in eukaryotic cells throughout the cytosol, in the nucleus and at the plasma membrane. Thus, despite evolutionary separation of billions of years, *Saccharomyces cerevisiae* and human actin have 87% amino acid sequence identity. F- and G-actin concentrations have been estimated to be as high as approximately 500 μM and 150 μM, respectively, in lamellipodia of mouse melanoma cells [27]. Association of *Pa*-ExoY with F-actin stimulates its activity more than 10,000 fold in vitro [17], which makes actin a potent cofactor for ExoY enzymatic activity. The functional specificities and virulence mechanisms of ExoY-like NC toxins and those related to the use of actin as cofactor remain to be further elucidated. For the *Yersinia* virulence factor YopO/YpkA, which also uses actin as cofactor in host macrophages, its interaction with G-actin activates its serine/threonine kinase activity [24]. To our knowledge, the extent of enzymatic stimulation by G-actin has not yet been defined. The targeting of YopO to G-actin is essential for its virulence mechanism in host cells as it also uses monomeric actin as bait to recruit and phosphorylate host actin polymerization-regulating proteins, resulting in deleterious forms of these ABPs that alters actin dynamics and consequently phagocytosis in host macrophages [24,28].

Second, cofactor-activated class II AC enzymes represent potent AC compared to other structurally-unrelated AC. Calmodulin-activated EF and CyaA have at least 40- to 100-fold higher AC activity than class III AC (tmAC or sAC), the most abundant ACs from bacteria to humans [29,30,31,32,33]. Actin-activated ExoY from *P. aeruginosa* (*Pa*-ExoY) or *Vibrio nigripulchritudo* (*Vn*-ExoY), an emerging marine pathogen infecting farmed shrimps, also exhibit a strong AC activity that is only slightly (about 10-fold) lower than that of activated EF and CyaA toxins [17,21].

Third, although originally identified as adenylate cyclase toxins, the cytotoxicity of NC toxins may be more complex than originally thought because their catalytic site may use nucleotide triphosphates (NTPs) other than ATP as substrates. Thus, in addition to their strong preference for ATP, the adenylate cyclase domains (AC domains) of EF and CyaA can also use CTP as a substrate to catalyze the synthesis of cCMP (cytidylyl-cyclase activity) and increase the amount of cCMP in host cells, albeit to a much lesser extent than the amount of cAMP [34,35,36].

*Pa*-ExoY appears to be even more complex. It exhibits a promiscuous NC activity with the following substrate preference in vitro: GTP > ATP ≥ UTP > CTP [21], and was found to increase cNMP levels in the order cUMP > cGMP > cCMP > cAMP in lung epithelial cells infected by an ExoY-expressing *P. aeruginosa* strain [35]. Thus, actin-activated *Pa*-ExoY displays a k_cat_ for cGMP synthesis that is approaching 1000 s^−1^ [17] and, therefore, within the same order of magnitude as the catalytic rates measured for cAMP synthesis for the structurally-related AC domain of CyaA [32] or EF [37] activated by calmodulin. In contrast, *Vn*-ExoY shows a strong preference for ATP as substrate and can use CTP with lower efficiency [21], thus behaving like EF and CyaA concerning its substrate specificity (Figure 2).

The cyclic pyrimidine nucleotides cytidine 3′,5′-cyclic monophosphate (cCMP) and uridine 3′,5′-cyclic monophosphate (cUMP) have now been unequivocally identified in mammalian cells, but the specific signaling functions and effectors regulated by these non-canonical cNMPs in cells are largely unknown and are only beginning to emerge (to see for recent reviews [44,45,46]). Thus, the potential interest of these toxins in modulating cCMP or cUMP levels in eukaryotic cells remains to be characterized in detail and represents an exciting area of future research [44,45]. To date, most of the cytotoxicity mechanisms and molecular signaling pathways affected in eukaryotic cells invaded by CyaA, EF, and ExoY NC toxins have been associated primarily to cAMP-dependent signaling pathways. The CyaA and EF enzymes, which have been studied for some time, are important models for understanding the broad spectrum of cytotoxicity that their calmodulin-triggered adenylate or nucleotidyl cyclase activity can cause in various eukaryotic cell types, including their numerous effects on the host cytoskeleton regulation.

## 3. Calmodulin (CaM)-Activated Bacterial Toxins

### 3.1. CyaA, an Adenylate Cyclase Virulence Factor from Bordetella pertussis

The Adenylate cyclase toxin, CyaA, is one of the virulence factors secreted by *Bordetella pertussis*, the bacterial pathogen responsible for the contagious disease, whooping cough [47]. CyaA is a 1706-residue-long protein secreted via a Type I secretion system (T1SS) that contains five distinct functional domains: an N-terminal catalytic AC domain, two consecutive domains, called translocation (T) and hydrophobic pore-forming (H) regions involved in interactions with membrane and translocation of the AC domain, an acylation region (A) that is acylated to convert proCyaA into an active toxin and a C-terminal RTX domain with low intrinsic hemolytic activity [48] (Figure 2).

**Figure 3:**
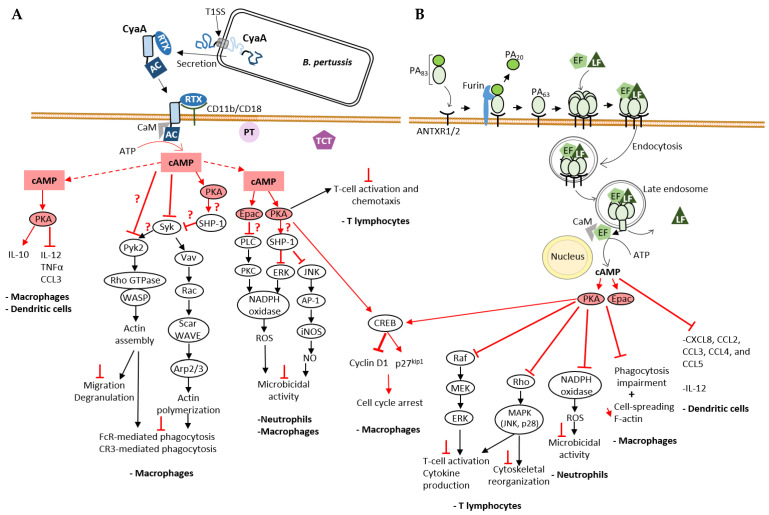
Mode of cell entry of CyaA and EF and their impact on cellular cAMP signaling cascades on immune cells. (**A**) cAMP-mediated processes disrupted by *B. pertussis* CyaA NC toxin. CyaA is one of the three major virulence factors that contribute to *B. pertussis* infection along with *pertussis* toxin (PT) and tracheal cytotoxin (TCT). A schematic of CyaA secretion through the T1SS and its cell entry mechanism, explained in detail in the text (Section 3.1), is shown here. Physiological signaling cascades are represented by black arrows for positive regulation, and the effects of bacterial toxins on signal transduction by red arrows for positive regulation, or red T-shaped bar for inhibition. A red question mark is added when the molecular mechanism is still unknown. CyaA-provoked cAMP accumulation disrupts the action of immune cells: CyaA disrupts the inflammatory response [54,55], inhibits both FcR- and CR3-mediated phagocytosis [42,56,57,58,59,60,61] and the microbicidal activity of these cells including the production of reactive oxygen species (ROS) [62,63]. (**B**) cAMP-mediated processes disrupted by *B. anthracis* EF NC toxin. EF and LF, two virulence factors secreted by *B. anthracis*, require the protective antigen (PA) for their cellular uptake as explained in the text (Section 3.3). Like CyaA, EF also interferes with the immune response and affects processes such as T cell activation [64,65,66,67], phagocytosis [42,68], cytokine [69] and ROS production [70], thus contributing to bacteria survival. Both CyaA and EF stimulate CREB phosphorylation leading to an increase in the expression of the CDK inhibitor p27Kip1 and a decrease in the amount of Cyclin D1, both of which cause cell cycle arrest [71].

CyaA binds, via its C-terminal RTX domain to the cell surface receptor CD11b/CD18 (CR3, αMβ2 integrin, or Mac-1) [49] of cells of the innate immune system, which are therefore natural targets of the toxin. Binding to receptor precedes CyaA interaction with membrane and insertion of the hydrophobic and translocation domains into the membrane, which allows the translocation of the AC domain into the submembranous space through the pore created by the hydrophobic domain (Figure 3A). Translocation of CyaA into cells lacking CD11b/CD18 receptors, such as erythrocytes or epithelial cells, occurs with a lower efficiency and remains to be elucidated in detail. In 2010, Eby et al. showed that CyaA translocates into polarized epithelial cells by interacting with membrane lipids across their basolateral membranes and with little or no translocation through apical membrane [50]. CyaA was also proposed to be delivered to epithelial cells through bacterial outer membrane vesicles (OMV) [51]. The cytotoxicity of CyaA is thought to be mainly due to its ability to increase intracellular cAMP in host cells [48]. It has been suggested that once translocated, CyaA remains associated to the plasma membrane [40,52]. In the host cell, CyaA becomes activated upon interaction with calmodulin (CaM) [9], a calcium sensor and signaling protein expressed in all eukaryotic cells and regulating many cellular processes. Structurally, CaM is composed of two globular domains (N- and C-terminal), each containing two calcium-binding sites connected by a flexible α-helix.

A recent study suggested that CyaA exists in at least two conformations, each with a different CaM binding affinity (Kd = 60 nM for lower affinity form and Kd = 0.06 nM for higher affinity form) and activation potential [32]. Once activated, both CaM-activated toxins, CyaA and EF, exhibit an AC activity that is about three orders of magnitude higher than that of ACs of host cells [37,53]. These toxins then produce supraphysiological amounts of cAMP, thereby interfering with intracellular signaling in infected cells.

### 3.2. Disruption of cAMP Signaling Pathway by CyaA and Its Role in Pathogenesis

By disrupting cAMP signaling, CyaA suppresses the initial responses of the innate immune system in the early stages of infection and promotes bacterial colonization, proliferation and dissemination. CyaA reprograms the expression of inflammatory cytokines and chemokines in immune cells: the toxin stimulates the production of anti-inflammatory cytokine IL-10 and inhibits production of pro-inflammatory cytokines IL-12, TNFα and chemokine CCL3 by macrophages and dendritic cells [54] (Figure 3A). Bryn et al., 2006 showed that secretion of TNFα, IL-12 and chemokines by monocytes was inhibited via the signaling pathways dependent on protein kinase A (PKA, also known as cyclic AMP-dependent protein kinase) [55].

Several studies have shown that CyaA inhibits phagocytosis. In this context, CyaA inhibits both FcR (Fc receptor) and CR3 (Complement Receptor 3)-mediated phagocytosis [58]. This effect is associated to actin cytoskeleton rearrangement and massive membrane ruffling in macrophages, which is triggered by the inhibition of RhoA and activation of cofilin, an actin-binding protein (ABP) that triggers severing and depolymerization of older filaments enriched in ADP-actin subunits [58]. In 2015, Osicka et al. investigated how CyaA inhibits CR3-mediated phagocytosis [60]. Binding of endogenous ligands to the CR3 (CD11b/CD18) receptor induces activation of Syk tyrosine kinase and downstream signaling involved in phagocytosis. CyaA exploits the physiological role of CR3 by binding to a different site than the I-domain used by endogenous ligands. In addition, CyaA-produced cAMP inhibits CR3-mediated Syk activation and its signaling pathway (Figure 3A). The Syk tyrosine kinase pathway also plays an important role in FcR-mediated phagocytosis. The effectors and downstream signaling proteins involved in the cAMP-mediated inactivation of Syk by CyaA remain to be further investigated. Since both Epac1, the cAMP-regulated guanine exchange factor (GEF) of the small GTPase Rap1 and PKA pathways inhibit FcR-induced phagocytosis in monocyte-derived macrophages [55], each or both of these cAMP effectors could be manipulated by CyaA to inhibit Syk. In the PKA pathway, CyaA AC activity leads to activation of the non-receptor phosphatase SHP-1, which is involved in Syk inactivation and inhibition of FcR-mediated phagocytosis [57] (Figure 3A). Recent findings by Hasan et al. have contributed to a better understanding of the effect of CyaA on the Syk tyrosine kinase signaling pathway and the various effector molecules [42]. In the THP-1 monocyte cell line, cAMP inhibits phosphorylation (activation) of both Syk and the Rho GTPase-specific GEF (RhoGEF) Vav, two mediators involved on opsonophagocytosis. Syk directly interacts and activates Vav by phosphorylation. Vav RhoGEF activation stimulates the transition of Rac-1 GTPase to its active GTP-bound state, which leads to actin cytoskeleton rearrangements necessary for phagocytosis [56] (Figure 3A). Rac-1-GTP activates the WASP family member, Scar/WAVE that induces recruitment and activation of the branched F-actin nucleation complex Arp2/3, which leads to assembly of actin filaments that drives pseudopods extension to enclose the opsonized prey [61]. CyaA also inhibits phosphorylation (activation) of the non-receptor protein tyrosine kinase Pyk2 [42]. Syk (and Src kinases) contributes to activation of Pyk2 that activates the Rho/WASP pathway of actin polymerization inducing actin cytoskeleton reorganization in immune cells required for phagocytosis and other processes such as migration and degranulation [59] (Figure 3A). CyaA can therefore disable phagocytosis by inhibiting phosphorylation of crucial signaling effectors involved in phagocytosis of opsonized targets. CyaA is also capable of inducing apoptosis of macrophages. Increase in cAMP levels by CyaA induces PKA-dependent activation of SHP-1 that will inactivate ERK1/2 and consequently lead to accumulation of the pro-apoptic protein BimEL. This accumulation could also be due to direct phosphorylation of BimEL by PKA. This leads to activation and insertion of Bax into mitochondrial membrane, ultimately leading to apoptosis [72]. Another consequence of CyaA’s AC activity is the cell cycle arrest of macrophages induced by different mechanisms. One of the proposed mechanisms is that activation of PKA by cAMP triggers an increase in CREB (cAMP response element-binding protein) phosphorylation and inhibition of ERK1/2 phosphorylation, which in turn leads to a decrease in Cyclin D1, contributing to the regulation of entry into S phase of cell cycle. CREB phosphorylation also leads to an increase in the CDK inhibitor p27Kip1, which impairs the G1-S cell cycle transition [71] (Figure 3A).

CyaA inhibits ROS production by neutrophils, which can be induced by different signaling cascades (FcR, CR3 receptors) (Figure 3A). CyaA-generated cAMP inhibits ROS production mainly by activating Epac signaling. Epac leads to inhibition of Phospholipase C (PLC) preventing activation of PKC, which normally contributes to the assembly and activation of NADPH oxidase and consequently to ROS production, which is required to kill invading pathogens [62]. PKA may also contribute to inhibition of ROS production by activating phosphatase SHP-1 [62]. This phosphatase could inactivate ERK thus preventing NADPH oxidase activation. Cerny et al., 2015 [63] showed that *B. pertussis* can survive NO-mediated intracellular killing by PKA-dependent activation of SHP-1 in response to CyaA-induced increase in cAMP levels. The proposed model of this mechanism states that SHP-1 leads to inactivation of the transcription factor AP-1, preventing iNOS expression and NO production.

CyaA also neutralizes the host’s adaptive immune defenses. This toxin impairs T-cell activation and chemotaxis via the cAMP/PKA signaling pathway [73] (Figure 3A). The MAP Kinase pathway, one of the effector cascades activated by TCR-signaling, plays a central role in T-cell activation [74]. CyaA inhibits TCR-dependent ERK (MAPK) activation in T lymphocytes [73] via cAMP-PKA signaling. The chemokine receptor (class A GPCRs) network plays a key role in T-cell trafficking and recruitment [75]: upon activation, chemokine receptors inhibit cAMP/PKA signaling and activate the MAP kinase pathway that regulates actin cytoskeleton rearrangement and cell motility [76]. By activating the cAMP-PKA-dependent pathway, CyaA inhibits CXCR4-dependent ERK1/2 phosphorylation, thereby inhibiting T-cell chemotaxis [73].

While immune cells appear to be the main target of CyaA, this AC toxin is also capable of entering and disabling non-immune cells. Recently, Angely et al. showed that CyaA induces in alveolar epithelial cells (cell line A549), a rearrangement of actin cytoskeleton as well as a reduction in focal adhesions leading to cell rounding and an increase in cytoskeleton stiffness, which consequently results in an inhibition of cell motility and impairment of their wound healing ability [77]. Hasan et al. also studied the role of CyaA in disrupting the epithelial barrier [78]. They showed that CyaA, through its cAMP signaling, disrupts the barrier function of bronchial cells (cell line VA10) by compromising the integrity of tight junctions. cAMP-signaling of CyaA also inhibits the production of proinflammatory cytokines (TNFα, IL-1β, IL-8) and induces actin cytoskeleton rearrangement in these epithelial cells. Reorganization of the actin cytoskeleton may be one of the elements contributing to the reduction in transepithelial electrical resistance (TEER) and barrier disruption.

### 3.3. Edema Factor (EF), an Invasive Adenylyl Cyclase from Bacillus anthracis

Edema Factor (EF) is a 92-kDa AC toxin secreted by *Bacillus anthracis*, the causative agent of anthrax. The two anthrax toxins, Edema toxin (ET) and Lethal toxin (LT), play a central role in the pathogenesis of anthrax and they derive from binary combinations of the three exotoxin components of the anthrax toxin complex secreted by this pathogen: Edema Factor (EF), Lethal Factor (LF) and peptide antigen (PA). ET is the combination of PA and EF, while LT is the combination of PA and LF. PA plays a key role in the internalization of the other two components into host cells (Figure 3B). The 83-kDa form of PA (PA83) binds the surface receptors ANTXR1 (or TEM8) [79] and ANTXR2 (or CMG2) [80], which are expressed by many cell types. PA83 is then cleaved by a furin-like protease releasing a 20-kDa domain (PA20) that allows the 63-kDa fragment of PA (PA63) to become heptameric while remaining bound to the receptor. PA63 heptamer, containing three binding sites, binds EF and LF competitively and the PA63-EF or PA63-LF complexes enter host cells through clathrin-mediated endocytosis. The acidic environment of the endosomes leads to a conformational change of the PA molecules, resulting in the insertion of a loop into the endosomal lipid bilayer and the formation of a pore [81]. EF or LF end up in perinuclear late endosomes and are then translocated into host cell through these pores [82]: EF remains bound to the membranes of late endosomes [41] and generates a perinuclear cAMP pool upon activation by calmodulin [8,40]. In contrast, LF diffuses into the host cell cytosol [41] (Figure 3B).

The structural architecture of EF is simpler than that of CyaA and can be divided into three modular domains: a 30-kDa N-terminal PA binding domain (PABD), a 43-kDa AC domain, and a C-terminal 17-kDa helical domain [6] (Figure 2). The N-terminal 30-kDa helical domain shows homology with the N-terminal domain of LF and is involved in the association of EF with protective antigen for host cell entry (Figure 3B). Both the AC and C-terminal helical domains are involved in the calmodulin interaction [37] (as detailed in Section 5.2), and EF AC domain displays 32% sequence identity with the 39-kDa AC domain of CyaA. Originally identified as a potent AC [8], EF may also exhibit modest cytidylyl-cyclase activity and consequently lead to an increase in cCMP in eukaryotic cells similar to CyaA AC domain [34,35,36]. The different localization of EF and CyaA in perinuclear late endosomes and at the subplasma membrane of infected cells, respectively, contributes to tune locally different subversion of cNMP-dependent host cell effectors and signaling pathways.

### 3.4. Disruption of cAMP Signaling Pathway by EF and Its Role in the Pathogenesis

Similar to the CyaA AC domain, the enzymatic activity of the EF AC domain contributes to disrupting host innate immune defenses and enabling successful invasion of host cells by *B. anthracis*. ET, the combination of PA and EF, has been shown to abolish production of pro-inflammatory cytokine IL-12 by dendritic cells and to disrupt their chemokine network (CXCL8, CCL2, CCL3, CCL4 and CCL5) [69], which may explain the effect of EF on T-cell activation [64] (Figure 3B).

ET inhibits phagocytosis of macrophages by activating both PKA and Epac signaling pathways [68] (Figure 3B). Inhibition of phagocytosis is accompanied by a reduction in macrophage spreading response and F-actin content. EF partially inhibits (by 50%) the phosphorylation of Syk, Pyk2 and Vav, effectors involved in opsonized phagocytosis, whereas CyaA completely inhibits the phosphorylation of these effectors in macrophages, as mentioned earlier [42]. One hypothesis to explain this difference between these two AC toxins is their different cellular localization [42]: the cAMP pool produced by CyaA at the subplasma membrane level and that of EF in the perinuclear region [40] may lead to a different modulation of the phagocytosis cascades.

Gray and Hewlett showed that ET, like CyaA, induces cell cycle arrest by impairing the G1-S transition via the same mechanism previously described for CyaA (Figure 3) [71].

ET also inhibits the oxidative burst (ROS) of neutrophils by inhibiting NADPH oxidase activity as a consequence of effects on the cAMP/PKA signaling [70] (Figure 3B). EF, like CyaA, inhibits the adaptive immune response of host cells. ET blocks T-cell activation in response to TCR stimulation [64]. ET-generated cAMP activates PKA, which in turn inhibits Raf [65], the first effector of the MAP kinase cascade, leading to the inactivation of the endpoint ERK1/2 [67] and thus inhibition of T-cell activation, cytokine production and proliferation (Figure 3B). PKA can also inhibit the GTPase Rho, resulting in inhibition of MAP kinase cascades, the endpoints of which are p38 mitogen-activated protein kinase (MAPK) and Jun N-terminal kinase (JNK), which normally contribute to inhibition of T-cell activation and cytoskeleton rearrangement [66]. EF inhibits T-cell chemotaxis by disrupting chemokine/chemokine receptor pathways [83]: this effect could be due to the disruption of MAP kinase cascades via activation of PKA by EF [66].

B-lymphocytes are also a target of ET because Edema toxin inhibits B cell migration and proliferation and modulates their cytokine production [84].

EF is also responsible for suppressive effects on endothelial cells. In these cells, both anthrax toxins, ET and LT, act synergistically to disrupt Rab-11-dependent trafficking, which is responsible for transporting important junctional proteins, such as the adhesion of protein E-cadherin to adherens junctions (AJ) in endothelial cells, resulting in the weakening of AJ [85]. A more recent study provides us with more mechanistic details of the effect of ET on the Rab-11-dependent transport: this toxin activates the PKA pathway and blocks interaction of the small GTPase Rab-11 with its effectors Rip11 and Sec15 [86]. Sec15 is an exocyst complex component recognizing vesicle-associated Rab GTPases (such as Rab11) and helps docking secretory vesicles and subsequently initiates its fusion with plasma membrane. Epac is also involved in the inhibition of this delivery process by preventing vesicle fusion at the cell surface. In 2011, Maddugoda et al., showed that ET disrupts the endothelium barrier also by inducing formation of transendothelial cell macroaperture (TEM) tunnels through the cAMP-signaling pathway [87]. Transcellular tunnels are known to contribute to particle exchange and are used by leukocytes during transcellular migration through endothelial cells [88]. Many bacterial pathogens secrete toxins that are capable of triggering and opening these tunnels in order to increase vascular permeability and promote bacterial dissemination. The actin cytoskeleton plays a key role in regulating endothelial barrier function and is often hijacked by bacterial pathogens. ET must hijack the actin cytoskeleton in order to cause the formation of these tunnels. Indeed, it has been shown that F-actin accumulates around TEM tunnels during the opening phase and a stiff actomyosin bundle encircles these tunnels [89] controlling their size. ADP-ribosylation and subsequent inhibition of the small GTPase RhoA by EDIN (Epidermal cell Differentiation INhibitor) toxin from *Staphylococcus aureus* leads to the disruption of actin cables and macroaperture formation in endothelial cells and mimics ET effects on endothelium disruption [90,91]. This suggests that ET may induce TEM tunnel formation and endothelial permeability, because PKA activated by cAMP can inhibit RhoA-mediated signaling

ET induces cytoskeleton rearrangement and cell rounding in mammalian kidney epithelial cell lines HEK193T and Y1 (mouse adrenal cortical cells) and the connective tissue cell line T24E (mouse embryonic fibroblasts) by generating cAMP and consequently activating PKA (but not Epac) [92].

## 4. Actin-Activated Bacterial Toxins

### 4.1. The Subfamily of ExoY-like Effector Proteins or Effector Modules Found in γ- or β-Proteobacteria

The Exoenzyme Y (ExoY/*Pa*-ExoY) was first identified in 1998 as a potent AC secreted through a type III secretion system (T3SS) by *Pseudomonas aeruginosa* (*P. a.*) [10]. *P. aeruginosa* is an opportunistic pathogen that causes severe infections in immune-compromised individuals. It is an important cause of nosocomial infections, such as pneumonia and infections of the urinary tract, wounds, burns and the bloodstream. *P. aeruginosa* also displays exceptional efficiency in colonizing the lungs of cystic fibrosis patients. The presence of the *exoY* gene in 98% of clinical *P. aeruginosa* isolates [12,13,14] suggests that it contributes significantly to the dissemination of the bacterium, but its role and underlying molecular mechanisms of virulence in various cell infections require further clarification [7,93]. The ExoY protein sequence encoded by different *P. aeruginosa* strains varies. Sequence variations can be without consequences on enzymatic activity, modulate it, or even nearly abolish it, as in the case of the ExoY sequences identified in highly virulent PA14 strains [94,95]. These variations, which may be a consequence of long-term evolution or short-term adaptation of *P. aeruginosa* strains, complicate the identification of the role of *Pa*-ExoY exotoxins. In a recent study, we examined the prevalence of ExoY activity in the largely diverse *P. aeruginosa* isolates of the international *P. aeruginosa* reference panel [95]. We found that ExoY GC activity is widespread among *P. aeruginosa* isolates, including those originating from cystic fibrosis patients, and that activity levels vary between strains (when measured in supernatants of bacterial cultures stimulated to secrete T3SS effectors by Ca++ chelation).

**Figure 4:**
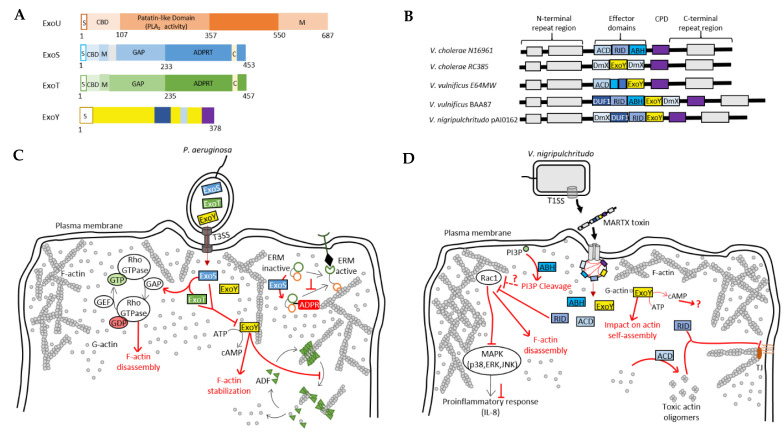
*Pseudomonas aeruginosa* and *Vibrio* pathogens co-inject into host cells several toxins/effectors that potentially act in a synergistic manner. (**A**) Domain organization of exotoxins secreted by the T3SS of *P. aeruginosa*. S, secretion signal; CBD, chaperone binding site; PLA2, phospholipase A2; M, membrane localization domain; GAP, GTPase activating protein activity; ADPRT, ADP-ribosyl transferase activity; C, cofactor binding site. (**B**) Domain organization of MARTX toxins secreted by many bacterial pathogens of the *Vibrio* genus [15]. (**C**) Model of the potential interplay between T3SS effectors of *P. aeruginosa*. ExoS and ExoT stimulate the inactivation Rho GTPases, which regulate actin cytoskeleton dynamics, thus leading to its disruption [11,96,97]. ExoS ADP-ribosylates and inactivates ERM proteins regulating many actin-based processes. ExoY recruitment to F-actin can disrupt the regulatory functions of cytoskeletal F-actin-binding proteins, such as ADF-mediated filament disassembly [17]. (**D**) Model of the potential interplay between the effector domains of MARTX toxins from *Vibrio* species. Cell entry mechanism of MARTX toxins is explained in detail in the text (Section 4.1). ABH, RID and ExoY modules all directly or indirectly impact actin cytoskeleton. RID-mediated inactivation of Rac1 leads to actin cytoskeleton disruption and inhibits, together with ABH, pro-inflammatory response [98]. ExoY may also impact actin self-assembly [17,99]. ACD induces the formation of toxic actin monomers [23,100] that will lead, together with RID, to a disruption of tight junctions (TJ).

Three other exotoxins are secreted by the *P. aeruginosa* T3SS: ExoS, ExoT and ExoU, which, like ExoY, must be unfolded to be secreted and pass through the T3SS syringe-like apparatus, and are activated by eukaryotic cofactors within host cells [11] (Figure 4A). Bacterial chaperone proteins may thus facilitate secretion of the effectors [11]. Within host cells, eukaryotic cofactors may additionally function as chaperones. ExoS and ExoT are two bifunctional toxins with a GTPase–activating protein (GAP) and an ADP-ribosyltransferase (ADPRT) activity. ExoU has a phospholipase A2 activity, inherent in a patatin-like domain that releases fatty acids from the sn-2 position of phospholipids. Most *P. aeruginosa* strains produce no more than three of the four T3SS-secreted effectors, with ExoS and ExoU mutually exclusive with exceptions.

More recently, ExoY-like NC modules have been found in some MARTX (Multifunctional-Autoprocessing Repeats-in-ToXin) or RTX toxins of γ-proteobacteria, including emerging human or animal pathogens. Functional ExoY-like NC enzymes in pathogens of the genus *Vibrio* are one example [15,17,21] (Figure 4B). MARTX/RTX toxins consist of a single polypeptide chain containing multiple effector domains separated by conserved boundary domains of repeated sequences. Effector domains vary between bacterial species or even between strains of the same species [15,16]. They are secreted by bacterial pathogens via a type I secretion system (T1SS) and bind the host cell membrane, where they form a pore using their N- and C-terminal domains (Figure 4B). The effector domains are then translocated through this pore. Once in the host cell, a cysteine protease domain (CPD) of the MARTX toxin is specifically activated and then releases multiple effector domains of this toxin, which subsequently disrupt key physiological functions in host cells.

**Figure 5:**
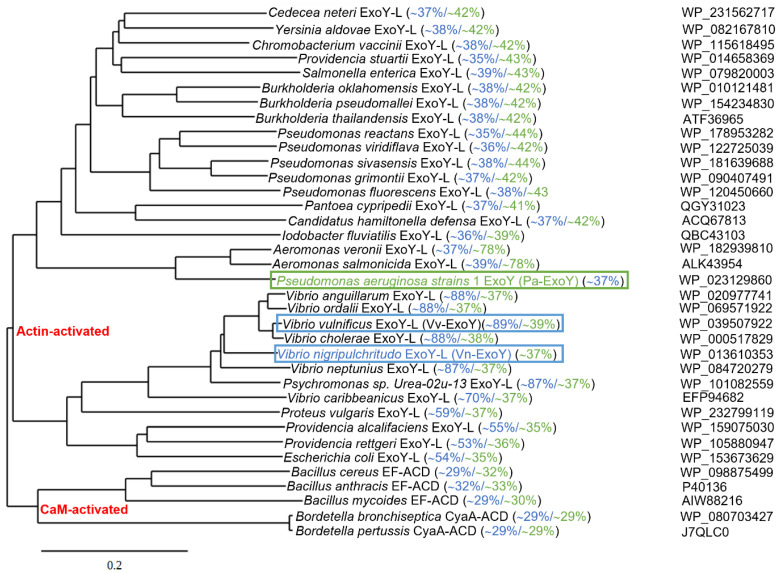
Phylogenetic tree of bacterial nucleotidyl cyclase toxins based on amino acid sequence comparisons. Alignment of the amino acid sequences of *Pa*-ExoY, Vn-ExoY and ExoY-like proteins/modules that are found in other γ- or β-proteobacteria and are potentially activated by actin, CaM-activated CyaA and EF and their homologues potentially activated by CaM, was made with Clustal Omega (using a neighbor-joining clustering method; http://www.ebi.ac.uk, accessed on 1 February 2022) [102]. Phylogenetic tree data obtained with clustal omega was then used to create the phylogenetic tree by using TreeDyn (www.phylogeny.fr) [103]. Pairwise sequence similarities (%) with *Pa*-ExoY and *Vn*-ExoY are displayed in green and blue, respectively (Sequence Identity And Similarity (SIAS) tool, http://imed.med.ucm.es/Tools/sias.html accessed on 21 May 2022). F-actin- and G-actin-activated ExoY toxins are framed in green and blue respectively. NCBI accession numbers of protein sequences are indicated on the right.

Uncharacterized ExoY-like toxins also exist as individual effector proteins in the genome of γ-proteobacteria other than *P. aeruginosa* [17,39]. Based on recent structures of complexes between G-actin or F-actin and ExoY-like toxins from bacteria of the genus *Vibrio* or *Pseudomonas* [39,101], we present in Figure 5 an updated phylogenetic tree of potential ExoY-like NC proteins encoded by sequenced bacterial genomes. The homologs that are listed contain at least part of the residues that are important for a proper recognition and activation by G- or F-actin [39,101]. Potential ExoY-like NC toxins that are mostly uncharacterized are found in various γ- or β-proteobacteria, including bacterial strains of the genera *Pseudomonas*, *Vibrio*, *Proteus*, *Aeromonas*, *Providencia* or *Burkholderia* (Figure 5). The amino acid sequences of these potential ExoY-like toxins show significant sequence variations overall and in their NC domain (down to only 35% sequence similarity between some NC domains, Figure 5). Some of these sequence variations might therefore result in ExoY-like NC homologues with different functional specificities.

Studies investigating the effects of deletion of the *exoY* gene in *P. aeruginosa* strains in different infection models have so far failed to reach a consensus [7,93]. In contrast, the ExoY NC effector domain of a MARTX proved essential for virulence of a *Vibrio vulnificus* (*V. v.*) strain that was associated with an outbreak of infections [15]. Some ExoY NC toxins could thus represent therapeutic targets against bacterial infections. Two ExoY-like NC homologs, which share only 38% amino acid sequence similarity, have so far been characterized more extensively at the level of their enzymatic specificities: the archetypal member of the ExoY-related subfamily *Pa*-ExoY, and an ExoY-like module present in the MARTX toxin of *Vibrio nigripulchritudo* (hereinafter referred to as *Vn*-ExoY) [17,21]. *V. nigripulchritudo* represents an emerging marine pathogen infecting farmed shrimps in New Caledonia and other regions in the Indo-Pacific, and a human seafood-associated pathogen causing severe wound and intestinal infections [15]. *Pa*-ExoY requires F-actin for maximal activation, is colocalized with F-actin in cells, and its binding to F-actin disturbs the regulated turnover of F-actin in vitro and in *Pa*-ExoY-transfected epithelial cells [17]. In contrast, *Vn*-ExoY is not activated by F-actin but is activated by G-actin, and the two ExoY-like NCs exhibit very different substrate specificity as mentioned above (Figure 2) [21]. The selective activation of ExoY-like homologues by G- or F-actin and their different substrate specificity may lead to different subcellular localizations in host cells and to the interference with very different signaling pathways. Actin-activated nucleotidyl cyclase virulence factors may therefore actually display a great variability of biological effects in infected cells despite sharing a common activator.

At the level of their structural organization, *Pa*-ExoY contains in addition to its conserved functional NC domain, a short, predicted intrinsically disordered (ID) N-terminal peptide (residues 1–19) (Figure 2). This extreme N-terminus is far from the interface with G- or F-actin [39,101]. The corresponding N-terminus of other T3SS exotoxins targets them to the T3SS. The N-terminal sequence of ExoY was thus proposed to fulfil the same signaling peptide function (Figure 4A) [11], but this remains to be demonstrated in *Pa*-ExoY. This bi-modular structural organization of *Pa*-ExoY with an ID extreme N-terminus may exist as well in some of the individual ExoY-like effector proteins, such as in the ExoY proteins from *Aeromonas salmonicida* (ID residues 1–34), *Providencia stuartii* (ID residues 1-28) or *Burkholderia pseudomallei* (ID residues 1–25). In the *P. aeruginosa* T3SS effector ExoS, the amino acids adjacent to the T3SS target peptide, namely residues 15–51, are thought to bind to the ExoS chaperone, SpcS [11]. No chaperone has yet been identified for *Pa*-ExoY.

For ExoY-like effector modules from MARTX/RTX toxins, the exact boundaries of ExoY-like effector modules after their CPD-mediated release remain poorly defined. Therefore, it remains to be seen whether or not released ExoY-like NC effector modules of RTX or MARTX toxins contain additional functional domains/peptides. All these aspects may affect the proper release and subcellular localization of ExoY-like NC toxins in host cells and need to be clarified by further studies.

### 4.2. Signaling Pathways Disrupted by Pa-ExoY

**Figure 6:**
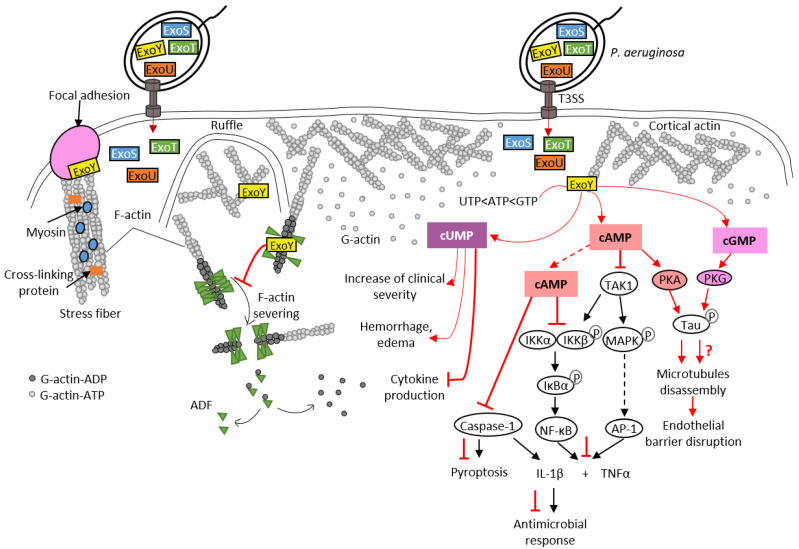
Pathogenicity of *Pa*-ExoY during *Pseudomonas aeruginosa* infection. *Pa*-ExoY (in yellow) is co-injected into host cells by the T3SS of *P. aeruginosa* along with three other exotoxins, ExoU, ExoT and ExoS, through the T3SS of *P. aeruginosa*. F-actin-activated *Pa*-ExoY can be recruited to the plasma membrane, to ruffles and to actin cables including stress fibers at vinculin focal adhesions [17]. *Pa*-ExoY alters F-actin turnover by inhibiting the cooperative binding and activity of ADF (Actin-Depolymerizing Factor). The *Pa*-ExoY-mediated increase in cUMP in mouse lungs coincided temporally with observed clinical alterations observed such as hemorrhage, edema and inhibition of cytokine production [93]. *Pa*-ExoY is likely to contribute to suppression of pro-inflammatory response in different cell types (epithelial cells, immune cells). *Pa*-ExoY AC activity inhibits production of pro-inflammatory cytokines (TNFα and IL-1β) by downregulating both NF-κB and AP-1 [104,105,107,108]. The cAMP-PKA signaling induces phosphorylation of the microtubule-associated protein Tau resulting in microtubule disassembly and endothelial barrier disruption [43,109,110,111]. The role of cGMP in endothelial permeability and barrier disruption remains unclear (red question mark). Physiological signaling cascades and toxin effects are shown in the same way as in Figure 3.

To date, *Pa*-ExoY is the only member of the actin-activated ExoY toxins that has been studied in detail at the cytotoxic level in different cell types. However, its role during bacterial infections is still debated [7,93]. Recent studies have shown that ExoY promotes virulence by modulating innate immune responses, as is the case with CyaA and EF NC toxins. *Pa*-ExoY reduces the expression of pro-inflammatory cytokine IL-1β in the monocyte cell line THP-1, and in infected epithelial cells (A549, BEAS-2B cell lines) [104] (Figure 6). This suppressive effect is mediated by the AC activity of *Pa*-ExoY, which delays phosphorylation of the IκB kinase (IKK) complex (IKK-αβ) and IκBα degradation, which in turn leads to delayed activation of NF-κB, a transcription factor that induces IL-1β expression. IL-1β maturation requires post-translational cleavage by caspase-1, which is also inhibited by *Pa*-ExoY [104,105]. The cAMP-mediated effect that inhibits the activation of caspase-1 is also responsible for the reduction in pyroptosis. Pyroptosis is a caspase-1-mediated cell death often triggered by microbial infections [106]. *Pa*-ExoY also inhibits secretion of pro-inflammatory cytokine TNFα [107] by THP-1 macrophage and A549 cell lines. *Pa*-ExoY inhibits production of these pro-inflammatory cytokines (TNFα and IL-1β) by blocking activation of the Transforming growth factor beta-Activated Kinase 1, TAK1 (Figure 6). In the TLR pathway, TAK-1 can activate both NF-κB and MAPK pathways (JNK, p38) to stimulate expression of pro-inflammatory cytokines [108].

Studies investigating the effect of deletion of the *exoY* gene in *P. aeruginosa* strains in different infection models have so far failed to reach a consensus [7,93], in part because expression of ExoY from plasmid with multiple copies compared to a single chromosomal copy can affect the outcome [93]. In epithelial cells, *Pa*-ExoY was previously found to potentially contribute to virulence by mediating bleb-niche formation via its AC activity, similarly to the ADP-ribosylation activity of ExoS, another T3SS-secreted toxin co-injected with ExoY [112]. *Pa*-ExoY-mediated bleb-niche formation may be due to several mechanisms: disruption of actin cytoskeleton leading to cell rounding [113] or microtubule breakdown as described below [109,110]. Further investigations are required to understand whether bleb-niche formation promotes bacterial trafficking and replication in cells.

In endothelial cells, *Pa*-ExoY induces a PKA-dependent phosphorylation of microtubule-associated protein Tau by generating a cytosolic cAMP pool, and leads to the formation of endothelial gaps [43,109,110,111] (Figure 6). The effects of *Pa*-ExoY-AC activity on the reorganization of the cortical actin rim, which is crucial for maintaining the barrier function of endothelial cells, is highly controversial. Disruption of the barrier by microtubule disassembly is accompanied by the reduction in cortical actin and stimulation of stress fiber formation, leading to gap formation [114]. However, Prasain et al. reported that cytosolic adenylate cyclase does not reorganize cortical actin into stress fibers but does induce gap formation and increases permeability [111]. Microtubule breakdown has been associated with pulmonary edema and impaired vascular repair after lung injury [115]. A recent study showed that *Pa*-ExoY plays an important role in converting antimicrobial amyloids into cytotoxicity prions composed of oligomeric amyloids, including beta amyloid (Aβ) and tau, thus inhibiting endothelial innate immune response [7,116]. Another study proposed that the *Pa*-ExoY-induced the accumulation of caspase-7 outside the cell leads to a cleavage of Aβ and/or tau oligomers that modulates their cytotoxic potency [117].

As previously mentioned, *Pa*-ExoY presents a broad substrate specificity and is able to use non-canonical nucleotides, such as UTP and CTP as substrates (Figure 2). A recent study suggested that the increase in cUMP induced by *Pa*-ExoY in infected mouse lungs could be responsible for *Pa*-ExoY-mediated pathophysiological changes such as hemorrhage, interstitial oedema formation and decreased production of proinflammatory cytokines (TNFα, IL-1β) [93].

### 4.3. Examples of cAMP-/cGMP-Signaling Directly Affecting the Actin-Cytoskeleton Regulation

The actin cytoskeleton is a frequent target of bacterial pathogens because its organization and regulation contribute to important cellular processes. The pathogen manipulates these processes in various ways to ensure successful infection [100,118,119,120]. Many actin-based cellular processes also involve cAMP or cGMP signaling pathways: for example, the cAMP- and cGMP-dependent kinases PKA and PKG regulate the activity of the actin elongation factor VASP (vasodilator-simulated phosphoprotein) [121,122,123], which plays a crucial role in many cellular processes, including the immune response. Bursts of cAMP have also been demonstrated to cause actomyosin redistribution, resulting in trajectory changes during T-cell migration [124]. The signaling cascade that controls these trajectory changes remains unclear. However, earlier research has supported the idea that the cAMP/PKA-signaling pathway controls actin-based cell migration [76]. The cAMP effector Epac, functions as guanine nucleotide exchange factor (GEF) for the small GTPase Rap1. It has been demonstrated that Epac-induced Rap-1 activation improves endothelial cell barrier function via an enrichment of cortical actin [125]. According to several studies, Rap1 appears to be important in the development of cell–cell junctions [126]. By disrupting cAMP signaling, bacterial AC toxins can directly disrupt the actin cytoskeleton and subvert it for their own benefit.

The utilization of actin as a cofactor is original among NC toxins, and since the discovery of this new subfamily of actin-activated ExoY-like toxins, one of the main interests is to understand why they use actin as a cofactor instead of calmodulin and what virulence specificities are associated with the use of this atypical cofactor. Could there be additional cytotoxicity induced by interaction with G- or F-actin independent of NC catalytic activity? *Pa*-ExoY can indeed alter F-actin turnover independently of its NC activity in *Pa*-ExoY-transfected epithelial cells [17]. Consistent with effects observed in cells, an inactive variant of *Pa*-ExoY inhibits the acceleration of F-actin disassembly induced by the regulatory ABP ADF (actin-depolymerizing factor)/cofilin in vitro [17] or can induce F-actin bundling in vitro [99]. Our recent biochemical studies of *Vn*-ExoY have also shown that its interaction with free or profilin-bound actin affects actin self-assembly dynamics in vitro independently of the NC activity of this toxin [101].

### 4.4. Possible Interplays between Actin-Activated NC Toxins and Co-Injected Virulence Factors

Bacterial pathogens secrete various virulence factors that may act sequentially and/or synergistically in infected cells. Identifying the role and precise mechanisms of actin-activated ExoY-like NC toxins will require further investigation of their cytotoxicity and virulence mechanisms, both alone and in combination with co-delivered toxins. Among the combinations of toxins to explore are bacterial cytoskeletoxins, all of which hijack the actin cytoskeleton. In *P. aeruginosa*, the T3SS effectors ExoS, ExoT and ExoY all directly or indirectly target the actin cytoskeleton [11,17]. ExoS and ExoT interact, for example, through their Rho GTPase-activating protein (RhoGAP) domain with Rho, Rac and Cdc42 Rho GTPases, which are central signaling regulators of actin cytoskeleton dynamics [127,128]. The ExoS and ExoT RhoGAP domains thus stimulate the transition from the active GTP-bound state to the inactive GDP-bound state of Rho GTPases, thereby disrupting the physiological regulation of the actin cytoskeleton (Figure 4C). This cytoskeleton disruption leads to cell retraction and endothelial barrier breakdown [96]. The ADPRT domain of ExoS is also involved in the disruption of the actin cytoskeleton of epithelial cells by inactivating small GTPases such as Ras, Rac and Cdc42, as well as proteins of the Ezrin, Radixin and Moesin (ERM) family. This inactivation occurs through the transfer of ADP-ribose to the target proteins [129] (Figure 4C). These ERM proteins contain an actin-binding C-terminal domain and are able to regulate many actin-dependent processes such as motility, phagocytosis and cell adhesion. By targeting these proteins, ExoS can therefore efficiently disturb the dynamics of the actin cytoskeleton of host cells. However, unlike in epithelial cells, *P. aeruginosa*-induced actin cytoskeleton collapse in endothelial cells depends only on the inactivation of the GTPases Rho, Rac and Cdc42 and not on the inhibition of ERM proteins [96]. ExoT also has an ADPRT domain, but unlike ExoS, it ADP-ribosylates other proteins such as the adaptor proteins CT10-regulator of kinase I (Crk-I) and Crk-II. Those proteins play a key role in signal transduction by mediating assembly of protein complexes. ADP-ribosylation of Crk-I prevents its association with components of focal adhesions, p130cas and paxillin, and thereby inhibits cell migration and extracellular matrix–cell interactions [130,131,132]. Genome-wide analysis of the response of a human lung epithelial cell line (A549) to the T3SS exotoxins of *P. aeruginosa* has shown that ExoS, T and Y elicit a different host transcriptional response when present in different combinations, suggesting a synergistic effect of these exotoxins [133]. This hypothesis is also supported by two more recent studies in which the authors suggest that there is likely to be an interplay between T3SS-secreted exotoxins, including *Pa*-ExoY. Huber et al. showed that *Pa*-ExoY, when injected alone, elevates cAMP levels in endothelial cells, which is not the case when this toxin is co-delivered with ExoS and ExoT [96]. Conversely, *P. aeruginosa* strains lacking ExoY are more cytotoxic to infected human lung epithelial cells than those secreting active ExoY, suggesting that ExoY may partially alleviate the cytotoxic effects of other *P. aeruginosa* virulence factors under certain conditions [95].

MARTX effectors may also act in synergy and their virulence could depend on the effector modules that they co-deliver. In *V. cholerae*, it has been demonstrated with that its MARTX toxin cytotoxicity is indeed the result of an interplay between some of its enzymatic effectors. First, the actin cross-linking domain (ACD) directly affects the actin cytoskeleton by inducing the formation of an isopeptide bond between actin monomers, resulting in actin oligomers that are not functional for actin polymerization [100,119] (Figure 4D). In T84 polarized epithelial cells, the delivery of ACD results in disruption of tight junctions, loosening the epithelial monolayer [16]. This loss of the trans-epithelial resistance (TEER) is also induced by a second effector domain, the Rho-inactivation domain (RID), which indirectly disrupts the actin cytoskeleton [16]. RID is a Nε-fatty acyltransferase that covalently modifies, by adding a fatty acid, the lysine residues in the C-terminal region of Rho GTPases such as Rac1, RhoA and Cdc42 [134]. Inactivation of Rac1 (and other Rho GTPases) by RID results in actin cytoskeleton disruption and suppresses the ACD-induced proinflammatory response (IL-8 and CXCL8) by blocking MAPK signaling pathways [98] (Figure 4D). A third enzymatic effector, the α/β hydrolase domain (ABH), is also involved in the inhibition of the proinflammatory response. ABH is a phospholipase A1 that cleaves phosphatidylinositol 3-phosphate (PI3P), resulting in altered lipid homeostasis. This process indirectly inhibits Rac1 as well and consequently, prevents the proinflammatory response, which is activated by ACD-induced cytoskeletal disruption [98] (Figure 4D). ABH has been shown to strongly increase the levels of activated Cdc42 when the effector is injected alone, whereas this effect is smaller when it is co-delivered with RID [98]. A potential interplay between RID, ACD and ExoY has been previously suggested [135]: RID and ACD, by stimulating actin depolymerization, could promote activation of ExoY. In *V. nigripulchritudo*, the ExoY effector module that is co-delivered with RID is indeed selectively activated by G-actin (Figure 4B,D) [135]. The effects of *Vn*-ExoY on the actin cytoskeleton need further elucidation. Taken together, these results suggest that the cytotoxicity of the MARTX effector modules, including those of ExoY-NC modules from MARTX/RTX, will most likely be modulated by the other effector domains co-injected into host cells.

## 5. Structural Models for the Activation and Catalytic Mechanisms of CaM- and Actin-Activated AC Toxins

Invasive bacterial NC toxins have no structural homology with the widespread class III of mammalian dimeric adenylyl or guanylyl cyclases (AC/GC) [37,38,136,137,138,139,140,141]. NC toxins may thus represent potential drug targets to specifically fight bacterial infections. Class III ACs have been more extensively characterized at the enzymatic level because they play an important role in many cellular processes and constitute relevant therapeutic targets for various human pathologies associated with cAMP-signaling dysregulation [142]. They represent interesting prototypes for analyzing how the active site of other enzymes of the NC family is built, modulated by activators, and may catalyze the cyclization reaction. Understanding their functional similarities and differences to class II ACs (i.e., NC toxins) may help determine how to specifically inhibit invasive bacterial NC toxins.

### 5.1. Models for the Activation of Class III ACs

NC, like other enzymes involved in phosphoryl transfer reactions, require one or more divalent metal cations as cofactors with nucleotide triphosphate substrates for catalytic activity [31,37,138,139,140]. The active site of class III tmAC/sAC is built at the interface of their conserved dimeric catalytic core architecture (corresponding to the cytosolic homologous catalytic domains C1a and C2a in the single protein chain of tmAC, Figure 1). Their two homologous catalytic domains are required to bind and properly stabilize the substrate ATP and metal ions, and to catalyze the cyclization reaction. The binding of GTP-Gαs heterotrimeric subunit for tmAC or bicarbonate for bicarbonate-responsive sAC to allosteric or adjacent second sites, respectively, activate their two homologous catalytic domains [33,136,138,143,144]. Structural studies suggest that tmAC or sAC undergo a transition from an open to a closed conformational state upon nucleotide binding, activation, and/or catalysis [33,136,138,143,144]. In tmAC, the AC C1/C2 heterodimer has not yet been captured in its inactive state to validate the different steps of transition between the inactive/open and active/closed states of C1 and C2. Models for the catalytic site activation are based on conformational changes observed between the structures of the tmAC C1-C2 tandem, activated and stabilized by GTP-Gαs and forskolin, with or without nucleotide ligands. ATP binding has been proposed to induce a collapse of the active site loops and sidechains of C1 and C2 around the substrate ATP, and the distant binding of Gαs to facilitate or stabilize the collapse to form a competent active site (detailed below in Section 5.3) [136,143]. In bicarbonate-responsive sAC, the binding of non-cyclizable ATP analogues (AMPcPP or Rp-ATPαS) with 1 or 2 metal ions was not sufficient for active site closure and stabilization of two metals in the active site [33]. The transient binding of bicarbonate has been proposed here to enable rearrangements of active site residues that favor metal binding, induce the open-closed transition in the presence of ATP and metals and/or support product release [33,138].

### 5.2. Models for the Activation of Bacterial NC Toxins (i.e., Class II ACs)

**Figure 7:**
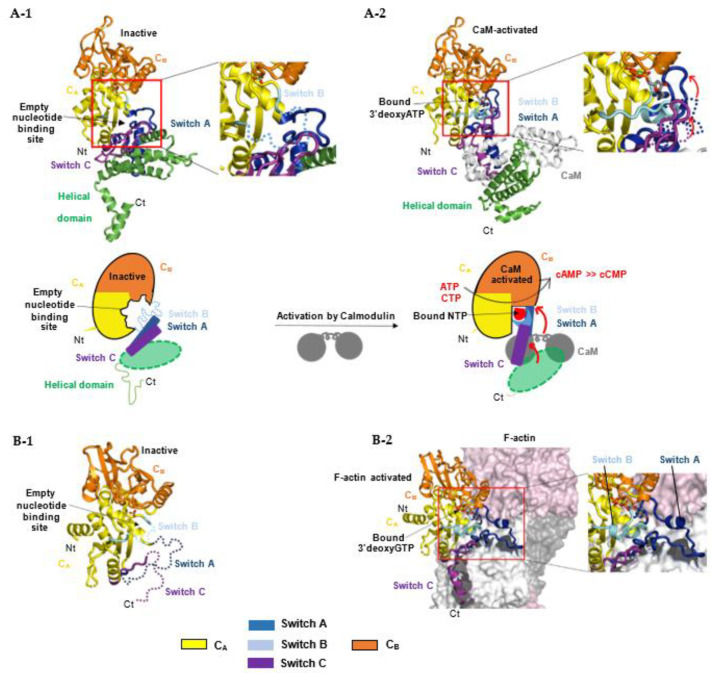
Allosteric activation mechanism of bacterial NC toxins by calmodulin (**A**) or F-actin (**B**) by stabilization of their nucleotide-binding pocket and catalytic site upon interaction with their cofactor. (**A**) The upper panel represents the different conformations of EF NC toxin during its activation process: (**A-1**) Free, inactive EF (PDB: 1k8t) [37]; in the absence of CaM, switch B is disordered (cyan dashed line). (**A-2**) CaM-activated EF with 3′dATP and a single metal ion Yb^3+^ in its active site (PDB: 1k90) [37]. CaM (white) inserts itself between the adenylate cyclase domain EF–AC domain (yellow) and the helical domain (green) of EF. The lower panel represents respective schematic models: CaM binding induces a large conformational movement of the interface regions switch A (dark blue) and C (purple) resulting in the stabilization of switch B, which contains residues that either bind ATP directly or stabilize catalytic residues in the groove between C_A_ and C_B_ domains [37]. The conformation of switch A and C regions in the absence of CaM are shown in blue and purple dashed lines respectively. (**B**) (**B-1**) Structure of free, inactive *Pa*-ExoY crystallized by limited in situ proteolysis (PDB: 5xnw) [38] with its switch regions, which are partially proteolyzed, dynamic or disordered in the crystal structure, shown in dashed lines. (**B-2**) Structure of F-actin-activated *Pa*-ExoY with 3′dGTP and a single metal ion Mg^2+^ in its active site solved by cryo-electron microscopy (PDB: code: 7p1g) [39].

For bacterial NC toxins, recent structural studies suggest that the structurally-related catalytic domains of EF, CyaA and ExoY-like NCs share a similar allosteric mechanism of activation by calmodulin (CaM) or actin, although these cofactors are very different in size and folding. Structural studies on CaM-activated EF and CyaA provided the first insights into how the cofactor binding triggers allosteric activation of their monomeric catalytic domain. The catalytic NC domain of EF, CyaA or ExoY-like toxins can be divided structurally into two subdomains, C_A_ and C_B_, with the catalytic site located in a groove at their interface (Figure 7A-1) [37,38,137]. In the inactive state of free EF, an important loop from its active site called switch B (residues 578-591 in EF, 299-311 in CyaA, 292-306 in *Pa*-ExoY) is disordered [37]. Switch B is located in EF subdomain C_A_ at the interface of C_A_ and C_B_ subdomains. It provides important ATP- and Mg^2+^-binding residues after activation (as described in Section 5.4) and has been proposed to act as a catalytic loop. The intrinsic disorder in switch B was therefore proposed to underlie the low basal NC activity of free NC toxins. Two neighboring regions of switch B, called switch A (residues 502-551 in EF and 199-274 in CyaA) and C (residues 630-658 in EF, 348-364 in CyaA) are also important in subdomain C_A_ (Figure 2 and Figure 7A-1) because they built the toxin-cofactor recognition interface of EF and CyaA with CaM [37,137]. In free EF, they both adopt a conformation away from switch B that is stabilized by the C-terminal helical domain of EF (residues 659-800). The latter is not present in CyaA or ExoY-like catalytic domains (Figure 2). Activation of EF or CyaA by CaM has been proposed to rely only on a CaM-induced movement of switch A and C towards the catalytic site [6,37,137]. This in turn stabilizes switch B and ATP-binding to promote a catalytically competent form of the enzyme (Figure 7A-2). In another study, a similar allosteric activation-by-stabilization mechanism was recently proposed for actin-activated NC toxins [39]. The structure of free *Pa*-ExoY was first solved using limited proteolysis of *Pa*-ExoY for crystallization [38]. In this inactive conformation of free *Pa*-ExoY, the absence of well-defined electron density for switches A, B and C suggested that these regions were either mobile, disordered and/or partially proteolyzed (Figure 7B-1). Additional insights have recently been provided by the cryo-EM structures of F- and G-actin-activated ExoY from *P. aeruginosa* and *V. vulnificus* (*Vv*-ExoY) determined at 3.2 and 3.9 Å resolution, respectively [39]. We also obtained different structural snapshots of *Vn*-ExoY interaction and activation mechanism by G-actin-ATP or -ADP at resolutions between 2 and 1.7 Å [101]. All these structures show that F- or G-actin-activated ExoY homologs use the same contacting regions with actin subunits as EF or CyaA with CaM, i.e., their switches A and C (Figure 7B-2), called sensor and anchor regions in [39], respectively. Molecular dynamics simulations on the structure of *Pa*-ExoY bound or unbound to F-actin suggest that its two switch regions, A and C, are highly flexible regions when the F-actin-activated structure of *Pa*-ExoY is unbound, and that changes at switch A and C are transmitted to the nucleotide-binding pocket [39].

All these structural studies, together with mutational and biochemical analyses [29,30,32,33,37,137,143,145,146,147], provide key insights into how the overall structures and catalytic sites of class III and class II AC are organized and modulated by their different activators to evolve towards catalysis. However, many important aspects of their catalysis remain debated or elusive, including the exact sequence of structural rearrangements during the inactive-to-active transition of their catalytic site, during substrate and metal ion binding, the cyclization reaction and product dissociation, or concerning the main reaction pathway(s) for catalysis and the role of AC residues and metal ions during each step of the reaction [138,148,149,150].

### 5.3. Models for the Catalytic Mechanism of Class III ACs

**Figure 8:**
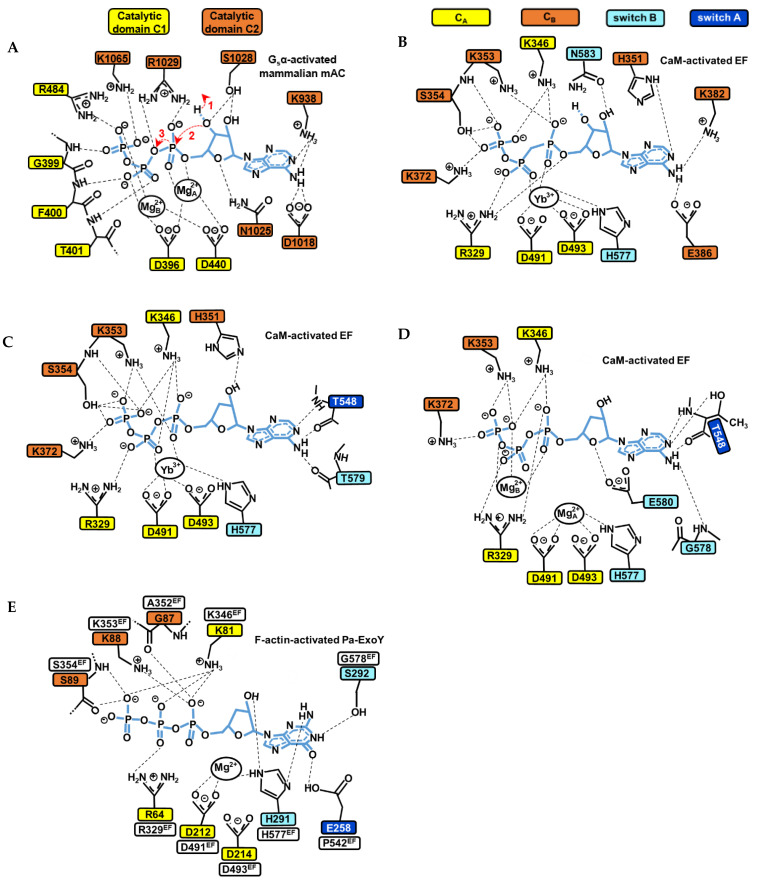
Structural model of the catalytic site of bacterial NC toxins and *mammalian* tm*AC* in the presence of ATP or GTP substrate analogues. (**A**) Simulated structural model of the catalytic site of the mammalian type V AC C1/type II AC C2 heterodimer in the presence of ATP based on the interactions seen with the non-cyclizable substrate analogue R_P_-ATPαS bound with two ion metals (PDB: 1CJK) [143]. The main steps 1, 2 and 3 of the cyclization reaction (to see text in Section 5.3) are indicated by red arrows. (**B**–**D**) The active site of CaM-activated EF structure with (**B**) the non-cyclizable ATP analogue AMPcPP and a single Yb^3+^ metal ion in PDB: 1S26 [31]. (**C**) The non-cyclizable ATP analogue 3′dATP and a single Yb^3+^ metal ion in PDB: 1K90 [37]. (**D**) 3′dATP and two Mg^2+^ metal ions in PDB: 1XFV [137]. (**E**) The active site of F- actin-activated *Pa*-ExoY with the non-cyclizable GTP analogue 3′dGTP and a single Mg^2+^ metal ion in PDB: 7P1G [39]. Thresholds used for detection of the interactions are those from PLIP (protein-ligand interaction profiler) web service [152].

Once AC enzymes are stabilized in a competent catalytic form by their cofactor(s), the catalytic reaction can take place. The following steps in the catalytic reaction were proposed in studies [136,138,148,149,150]: (1) deprotonation of the 3′ hydroxyl group (3′OH) of ATP ribose (Figure 8A); (2a) in-line nucleophilic attack of activated 3′OH on α-phosphate; (2b) stabilization of the transition state at the α-phosphate, most likely a pentavalent phosphate intermediate; (3) product formation and stabilization of the new negative charges on the pyrophosphate leaving group; and possibly (4) efficient release of the products to reinitiate the reaction. How the catalytic site of cofactor-bound NC toxins is tuned for and contributes in each of these different steps will require further investigations.

Class III tmAC/sAC use a two-metal ion catalytic mechanism, where the substrate ATP is associated to two metal ion cofactors (Mg^2+^ or Ca^2+^) for catalysis [33,138,143]. The cyclization reaction catalyzed by class III ACs can follow different reaction pathways, including a stepwise associative, stepwise dissociative or concerted mechanism [148]. In tmAC, the active site closure involves an inward collapse of several structural elements of C1 and C2 towards the active site, which decreases the distance between the purine-binding pocket and the phosphate-binding loop. This allows a simultaneous binding of the purine and phosphate moiety of ATP in the catalytic site [136,143,151]. The coordination of the ribose and adenine base is then provided by residues from C2, that of metals by residues from C1 and that of the phosphates by residues from both C1 and C2 (Figure 8A).

Several conserved residues and interactions in class III tmACs and sAC have been proposed to be essential for catalysis and can serve as models to study the catalytic site of NC toxins. The two metal ions are anchored by two aspartates (D396 and D440 in type V AC C1, referred as D396-D440^tmAC-VC1^) that are invariant among both class III and II ACs. In human sAC, the aspartate equivalent to D440^tmAC-VC1^, which is D99 (D99^sAC-C1^), is reoriented by bicarbonate transient binding allowing ion site formation [138]. The two metals are referred to as metal ion A and B. Metal ion A is localized closer to the ribose and coordinates the α-phosphate, whereas metal ion B coordinates the γ- and β-phosphates, and sometimes also the α-phosphate [138,143,148]. Ion A has been proposed as being particularly important for coordinating and polarizing the 3′OH to facilitate its deprotonation, ion B for substrate binding and stabilizing the conformation of the triphosphate moiety. In a computational investigation, both contributed to the steps of proton and phosphoryl transfer in the proposed most probable reaction path [148]. For the ribose moiety, the active site closure leads Asn1025^tmAC-IIC2^ (Asn412C2^sAC-C2^/Asn1146^cyanobacterial-sAC-C2^ in human and cyanobacterial sAC, respectively) [33,138,143] to form a hydrogen bond with ribose ring oxygen O4’, which is believed to be important for the correct positioning of the ribose during catalysis. For the triphosphate moiety, the collapse brings conserved positively charged residues close to the substrate (Arg1029^tmAC-IIC2^, Lys1065^tmAC-IIC2^ and Arg484^tmAC-VC1^, Figure 8A) that help, together with metal ions A and B, to bind the negatively charged triphosphate moiety of ATP, stabilize the transition state of the reaction and neutralize the negative charge on the PPi leaving group [33,138,139,141,143,148,153]. A computational study based on the structure of activated C1-C2 catalytic tandem of tmAC bound to ATPαS and 2 metal ions [143] provided valuable insights into the most probable catalytic mechanism and role of C1 and C2 residues and metals during different steps of the reaction [148]. For the initial deprotonation of 3′OH, the substrate ATP itself has been proposed to function as a general base, with water-mediated transfer of the 3′H to the γ-phosphate [148]. The analysis suggests that Mg^2+^_A_ is important for coordinating and polarizing the 3′OH of ATP to facilitate its deprotonation. Lys1065^tmAC-IIC2^ (Lys451^sAC-C2^/Lys1184^cyanobacterial-sAC-C2^), which coordinates the β- and γ-phosphates in the initial enzyme/substrate state (Figure 8A), may play a significant role in catalyzing this first step of water-mediated transfer of the 3′H to the γ-phosphate. In addition to K1065, the triphosphate coordination by D396D440^tmAC-VC1^, Mg^2+^_A_ and Mg^2+^_B_ are also critical for the first and subsequent steps. Proton transfer is indeed followed by changes in coordination of the two metal ions and in the conformation of ATP, before a proposed concerted phosphoryl transfer step occurs. Arg1029^tmAC-IIC2^ (Arg416^sAC-C2^/Arg1150^cyanobacterial-sAC-C2^), which coordinates the α- and β-phosphates in the initial enzyme/substrate state (Figure 8A), was eventually proposed to play a significant role in catalyzing the phosphoryl transfer [148].

### 5.4. Models for the Catalytic Mechanism of Bacterial NC Toxins

The catalytic mechanism of CaM-activated class II ACs toxins remains more debated because the structures of CaM- or F-actin-activated NC toxins with substrate analogues show different arrangements of their active site concerning the number of ions present, their position and coordination geometries and the conformation of substrate analogues, as illustrated in Figure 8B–D [31,37,39,137]. The competent conformation for the cyclization reaction of the active site of active NC toxins thus remains equivocal, and to date appears to be only remotely related to that of class III adenylate cyclases. Structures of CaM-activated EF bound to non-cyclizable ATP analogs have been solved with one, two (Figure 8B–D) [31,37,137] or a mixture of one and two metal ions with cAMP and PPi [31]. For actin-activated ExoY NC toxins, the recent cryo-EM structure of F-actin-activated *Pa*-ExoY shows the toxin active site filled with a non-cyclizable GTP analog associated with a single metal ion [39] (Figure 8E). Whether actin- or calmodulin-activated NC toxins catalyze the cyclization reaction in the same way and with how many ions, and what the nature and role of their catalytic base is and if their catalytic reaction differs from that of class III ACs with their reaction pathway, await further investigations. Computational studies have suggested that EF may efficiently catalyze the cyclization reaction with one or two metal ions [149,150], using a quasi-concerted catalytic mechanism with two Mg^2+^ [149], or highly associative mechanisms with one or two Mg^2+^ [150]. It has been proposed that the two-metal catalytic mechanism involves metal-assisted transfer of O3′ proton to the bulk aqueous solution [149]. Alternatively, the one-metal catalysis may rely on a high conformational flexibility and the presence of His351 in EF active site [150]. His351 can coordinate the 3′OH of ATP [37] (Figure 8C) and may act as general base to initiate its deprotonation in the EF active site [37,149,150].

The large heterogeneity in the active site of the structures described above prevents the precise identification of the role of residues in the catalytic reaction. However, these studies allow the identification of some common elements in the active site interactions of NC toxins and class III ACs. As in class III ACs, interactions with the phosphate, ribose and purine groups of the substrate and metals are distributed between different subdomains of the NC toxin that undergo conformational changes, some of which occur upon cofactor binding. The switch B interactions with the substrate analogs and metals are the most conserved, which confirms switch B importance for cofactor-mediated stimulation and catalysis [6]. In the activated conformations of EF and *Pa*-ExoY shown in Figure 8B–E, the switch B is always interacting with a metal ion in the active site, the ribose and/or purine base. The metal, which is difficult to assign as metal A or B, is coordinated by the conserved His577 of EF switch B or the equivalent H291 of *Pa*-ExoY. Mutating EF His577 to Ala or Asn causes a 170-fold reduction in EF catalytic activity [37]. Another notable conserved residue of switch B is Asn583^EF^ or Asn297^Pa-ExoY^. Mutating Asn583 to Ala, Gln or His in EF switch B causes at least a two-fold reduction in EF catalytic activity, highlighting the importance of the precise coordination of the Asn583 side chain [37]. This is reminiscent of the importance of Asn1025^tmAC-IIC2^ in class III AC for the ribose correct positioning during catalysis. However, the interaction of this conserved asparagine of switch B (Asn583^EF^/Asn297^Pa-ExoY^) with the ribose of the ATP analog is different from that provided by Asn1025^tmAC-IIC2^, and versatile in the active site of CaM- or F-actin-bound NC toxins. Its interaction with the ribose emerges clearly only in the structure of CaM-bound EF with AMPcPP (Figure 8B). The interactions with the purine base are specifically provided by C2 residues in tmAC, whose position in the class III AC catalytic center is modulated by the binding of activators and/or substrate. Here, these interactions are provided either by switch A that is indeed recognized and rearranged by the cofactor (in blue in Figure 8B–E), or by the C_B_ subdomain (in orange in Figure 8B), whose conformation does not depend on the cofactor in a trivial way, except perhaps through an allosteric communication still to be defined. The two invariant aspartates (D491-D493^EF^/D212-D214^Pa-ExoY^) that anchor one or two metals in the active site are provided by C_A_ subdomain, which also stabilizes the phosphate moiety via a conserved positively charged residue (R329^EF^/R64^Pa-ExoY)^, as C1 in tmAC. Conserved positively charged residues around the substrate that may mimic the role of Arg1029^tmAC-IIC2^, Lys1065^tmAC-IIC2^ and Arg484^tmAC-VC1^ in tmAC (Figure 8A) are distributed between C_A_ and C_B_ subdomains of NC toxins (Figure 8B–E). A notable conserved interaction with the α-phosphate is provided by the conserved Lys346^EF^/Lys81^Pa-ExoY^, which belongs to the conserved ATP-binding motif I of NC toxins [10] in their C_A_ subdomain. This conserved lysine may act like Arg1029^tmAC-IIC2^ in class III AC (role in catalysing the phosphoryl transfer step) [148]. The conserved lysine in motif I (Lys346^EF^/Lys81^Pa-ExoY^) is frequently mutated to Met to inactivate the NC toxins [10,146] for cell biology studies. However, full inactivation of the NC toxins requires a double mutation in motif I, K346M-K353M in EF or K81M-K88M in *Pa*-ExoY [21], suggesting that this second conserved Lys is important in other steps of the cyclization reaction. The coordination provided by Lys353^EF^ or Lys88^Pa-ExoY^ is more versatile in the available structures, where the lysines coordinate either the α- and/or γ-phosphates (Figure 8B–E). This second conserved lysine belongs to C_B_ subdomain, which was observed to rotate as a rigid body relative to C_A_ between the structures of CaM-bound EF and CyA [137]. The lysine belongs therefore to another mobile region of the active site and could act as Lys1065^tmAC-IIC2^ in class III ACs in the hypothesis of a two-ion catalytic mechanism. In the hypothesis of a single-ion catalytic mechanism, its role remains to be determined. It also remains to be better understood how NC toxins can display higher catalytic rates than class III ACs [6,17,30,31,32,33,37,144,150]. It has been proposed that the greater flexibility of their active site as illustrated in Figure 8 allows the sampling of lower-energy conformations and might represent an entropic advantage for catalytic efficiency [150]. Further investigations are needed to clarify all outstanding questions raised in this paragraph regarding the active site dynamics and catalytic mechanism(s) of activated NC toxins.

## 6. Development of Selective Inhibitors of Bacterial AC Toxins

Structural differences between class II bacterial AC toxins and class III mammalian AC (tmAC or sAC), e.g., differences between their catalytic sites, play an important role in the development of selective inhibitors of CaM-activated EF and CyaA toxins [154]. Inhibitors that target and block the catalytic site of bacterial toxins have been sought. P-site inhibitors have been identified early, first as small molecules capable of inhibiting cAMP production in cells [155]. P-site inhibitors are inhibitors that require the presence of an intact adenine ring, and in addition, for efficient inhibition a polyphosphate tail or pyrophosphate [144,151]. They have been found to progressively bind directly to tmAC by targeting one of the two distinct adenosine-sensitive sites of their purified C1-C2 tandem, which was designated as P-site [155]. By binding to the catalytic site of tmAC, these adenosine nucleotide analogues (3′-nucleoside mono-, di-, -triphosphates) decrease the AC activity through an uncompetitive or competitive inhibition mechanism with respect to the substrate ATP or the products cAMP and pyrophosphate, respectively [151]. However, P-site inhibitors decrease the AC activity of CaM-activated toxins EF and CyaA in a less potent manner [156]. The most potent inhibitor of EF (Ki = 27 nM in vitro) and CyaA (Ki = 25 nM) is adefovir diphosphate (PMEApp) [157,158], the active metabolite of adefovir dipivoxil, an approved drug used for therapy in the treatment of chronic hepatitis B. This molecule also competes with the binding of natural substrate ATP to EF or CyaA displaying a stronger affinity for their AC domain than the ATP substrate [157]. A major issue of the inhibitors targeting the catalytic site is the difficulty to develop molecules that are highly selective of bacterial AC toxins relative to Class III ACs.

Inhibitors targeting the EF- or CyaA-CaM interface have also been developed. In a morphology-based assay conducted by the group of W.J. Tang, in which more than 10,000 compounds were tested [159], 4-[4-(4-nitrophenyl)-thiazolylamino]-benzene-sulfonamide was found to inhibit the interaction of CaM with both CyaA and EF (IC50 of 10 μM for EF) [159]. This molecule binds to the helical domain of EF and the interface between this domain and the catalytic core of EF therefore disrupting EF–CaM interaction. One of the drawbacks of this chemical is the fact that it presents substantial cytotoxic effects, making it unsuitable for therapeutic usage [159].

Targeting the catalytic site and the EF/CaM interaction were the two major approaches used in the development of inhibitors against CaM-activated toxins. A more recent approach is to develop allosteric inhibitors that bind to sites other than the catalytic and CaM contact sites that are unique to these bacterial AC toxins. In one study, modeling the transition between inactive and CaM-activated EF conformations [160] allowed the identification of potential sites that could impact EF activation. A series of thiophene derivatives were identified, of which the most potent compound inhibited EF activity (IC50 = 2 μM), thus less effectively than PMEApp [160]. These bacterial structurally-related AC toxins of the class II could represent potential drug targets to combat various bacterial infections. For these compounds to be used in clinic, many issues need to be solved, including their selectivity relative to all mammalian AC and GC enzymes, toxicity and solubility [161].

It will be interesting to determine whether some of these compounds developed against CaM-activated toxins can also lead to inhibition of actin-activated ExoY-like toxins found in bacteria that are important human and animal pathogens. Successful in establishing infections of immunocompromised patients and difficult to treat due to multidrug resistance [14], *P. aeruginosa* ranks among the WHO’s top priority pathogens. *P. aeruginosa* strains with a functional T3SS cause a higher bacterial burden and mortality in acute respiratory infections [162]. Among *the* T3SS effectors of *P. aeruginosa*, ExoY is one of the two most prevalent exotoxins with ExoT encoded in the genomes of clinical (nosocomial or chronic CF infections) and environmental *P. aeruginosa* isolates [12,13,14], suggesting an important role of *Pa*-ExoY in bacterial dissemination and survival. As outlined above, recent studies suggest that *Pa*-ExoY contributes to deteriorate the host immune response [93,104,107,116] and to acute lung infections [93,115,116,117]. ExoY effector proteins/modules are present among the virulence arsenal of various γ- or β-proteobacteria (Figure 4 and Figure 5), which are responsible for various infections in human or animals. In wound infections by *Vibrio vulnificus*, a potentially life-threatening emergent human pathogen, the MARTX effector module *Vv*-ExoY MARTX has been found to be essential for bacterial virulence [15]. An ExoY-like toxin is also found in *Burkholderia pseudomallei* (Figure 5). The latter is the causative agent of melioidosis, which affects both humans and animals and is considered as a potential bioterror agent [163]. There is thus an urgent need to explore the potential of actin-activated NC toxins as drug target against *P. aeruginosa*, *Vibrio*, *Providencia*, *Proteus* or *Burkholderia* strains that can secrete ExoY-like toxins and are involved in severe chronic, nosocomial, wound- or burn-associated infections. In the future, inhibitors of CaM- and actin-activated toxins may be used as a potential adjunctive therapy for various infectious diseases.

## 7. Conclusions

Although important eukaryotic signaling pathways manipulated by bacterial nucleotidyl cyclase toxins have been identified, there is still much to be learned about their spectrum of cytotoxicity, the details of the effectors and signaling pathways involved, as well as virulence mechanisms of different bacterial species and biotypes in different eukaryotic cell types. Additional biochemical, structural and computational investigations are also required to understand how the catalytic machinery and active sites of bacterial NCs work in detail at each step of the reaction and in a more efficient and toxic manner compared to mammalian adenylate and guanylate cyclases. These elements will be important to search for specific inhibition mechanisms against bacterial NC toxins that prove to be essential for bacterial virulence [15]. These toxins represent sophisticated offensive weapons available to pathogens. They may also represent interesting molecular tools to investigate how canonical and non-canonical cNMPs regulate basic cellular and metabolic processes in different subcellular compartments. Interestingly, a recent study has shown that cCMP and cUMP are produced in Gram-negative bacteria following phage infection to mediate bacterial immunity against the bacterial viruses [164]. Studies on the virulence mechanisms of bacterial NC toxins in various bacterial infections and eukaryotic cells may thus contribute to the identification of signaling pathways that depend on these non-canonical secondary messengers.

## Data Availability

Not applicable.

## Abbreviations

3′dATP	3’-Deoxyadenosine-5’-Triphosphate or Cordycepin triphosphate
3′dGTP	3’-Deoxyguanosine-5’-Triphosphate
ABH	α/β hydrolase domain
AC	Adenylate Cyclase
ACD	Actin cross-linking domain
AMPcPP	Adenosine-5’-[(α,β)-methyleno]triphosphate
CaM	Calmodulin, a signaling Ca^2+^-binding protein
cAMP	3′,5′-cyclic adenosine monophosphate
cCMP	3′,5′-cyclic cytidine monophosphate
cGMP	3′,5′-cyclic guanosine monophosphate
cNMPs	3′,5′-cyclic nucleotide monophosphates
CREB	cAMP response element-binding protein
cUMP	3′,5′-cyclic uridine monophosphate
EDIN	Epidermal cell Differentiation INhibitor
EF	Edema Factor from *Bacillus anthracis*
ERK	Extracellular signal-regulated kinase
ExoY	Exoenzyme Y
ET	Edema toxin from *Bacillus anthracis*
GAP	GTPase-accelerating protein
GC	Guanylate Cyclase
GEF	Guanine exchange factor
GPCR	Transmembrane G Protein-Coupled Receptors
JNK	Jun N-terminal kinase
LT	Lethal toxin from *Bacillus anthracis*
MAPK	Mitogen-activated protein kinase
MARTX	Multifunctional-autoprocessing repeats-in-toxin toxin
MEK	Mitogen-activated protein kinase
NC	Nucleotide Cyclase
*P. a.*	*Pseudomonas aeruginosa*
PA	Protective antigen exoprotein from *Bacillus anthracis*
PDE	Phosphodiesterase
PKA	Protein kinase A, also known as cyclic AMP-dependent protein kinase
PKG	Protein kinase G, also known as cyclic GMP-dependent protein kinase
PMEApp	Adefovir diphosphate or 9-(2-(phosphonomethoxy)ethyl)adenine diphosphate
PPi	Pyrophosphate
RID	Rho-inactivation domain from MARTX/RTX toxin
ROS	Reactive oxygen species
Rp-ATPaS	Rp isomer of Adenosine-5’-(α-thio)-triphosphate
RTX	Repeats-in-toxin exoproteins
sAC	Soluble class III Adenylate Cyclase
T1SS	Bacterial type I secretion system
T3SS	Bacterial type III secretion system
TAk1	Transforming growth factor beta-Activated Kinase
tmAC	Transmembrane class III Adenylate Cyclase
*V. n.*	*Vibrio* *nigripulchritudo*
*V. v.*	*Vibrio* *vulnificus*

## References

[1] McDonough K.A., Rodriguez A. (2011). The myriad roles of cyclic AMP in microbial pathogens: From signal to sword. Nat. Rev. Microbiol..

[2] Mangmool S., Kurose H. (2011). G(i/o) protein-dependent and -independent actions of Pertussis Toxin (PTX). Toxins.

[3] Katada T. (2012). The inhibitory G protein G(i) identified as pertussis toxin-catalyzed ADP-ribosylation. Biol. Pharm. Bull..

[4] Brito G.A., Souza M.H., Melo-Filho A.A., Hewlett E.L., Lima A.A., Flores C.A., Ribeiro R.A. (1997). Role of pertussis toxin A subunit in neutrophil migration and vascular permeability. Infect. Immun..

[5] Ahuja N., Kumar P., Bhatnagar R. (2004). The adenylate cyclase toxins. Crit Rev. Microbiol..

[6] Tang W.J., Guo Q. (2009). The adenylyl cyclase activity of anthrax edema factor. Mol. Asp. Med..

[7] Balczon R., Morrow K.A., Zhou C., Edmonds B., Alexeyev M., Pittet J.F., Wagener B.M., Moser S.A., Leavesley S., Zha X. (2017). Pseudomonas aeruginosa infection liberates transmissible, cytotoxic prion amyloids. FASEB J..

[8] Leppla S.H. (1982). Anthrax toxin edema factor: A bacterial adenylate cyclase that increases cyclic AMP concentrations in eukaryotic cells. Proc. Natl. Acad. Sci. USA.

[9] Wolff J., Cook G.H., Goldhammer A.R., Berkowitz S.A. (1980). Calmodulin activates prokaryotic adenylate cyclase. Proc. Natl. Acad. Sci. USA.

[10] Yahr T.L., Vallis A.J., Hancock M.K., Barbieri J.T., Frank D.W. (1998). ExoY, an adenylate cyclase secreted by the Pseudomonas aeruginosa type III system. Proc. Natl. Acad. Sci. USA.

[11] Hauser A.R. (2009). The type III secretion system of Pseudomonas aeruginosa: Infection by injection. Nat. Rev. Microbiol..

[12] Bradbury R.S., Roddam L.F., Merritt A., Reid D.W., Champion A.C. (2010). Virulence gene distribution in clinical, nosocomial and environmental isolates of Pseudomonas aeruginosa. J. Med. Microbiol..

[13] Feltman H., Schulert G., Khan S., Jain M., Peterson L., Hauser A.R. (2001). Prevalence of type III secretion genes in clinical and environmental isolates of Pseudomonas aeruginosa. Microbiology.

[14] Fakhkhari P., Tajeddin E., Azimirad M., Salmanzadeh-Ahrabi S., Abdi-Ali A., Nikmanesh B., Eshrati B., Gouya M.M., Owlia P., Zali M.R. (2022). Involvement of Pseudomonas aeruginosa in the occurrence of community and hospital acquired diarrhea, and its virulence diversity among the stool and the environmental samples. Int. J. Environ. Health Res..

[15] Ziolo K.J., Jeong H.G., Kwak J.S., Yang S., Lavker R.M., Satchell K.J. (2014). Vibrio vulnificus biotype 3 multifunctional autoprocessing RTX toxin is an adenylate cyclase toxin essential for virulence in mice. Infect. Immun..

[16] Dolores J.S., Agarwal S., Egerer M., Satchell K.J.F. (2015). Vibrio choleraeMARTX toxin heterologous translocation of beta-lactamase and roles of individual effector domains on cytoskeleton dynamics. Mol. Microbiol..

[17] Belyy A., Raoux-Barbot D., Saveanu C., Namane A., Ogryzko V., Worpenberg L., David V., Henriot V., Fellous S., Merrifield C. (2016). Actin activates Pseudomonas aeruginosa ExoY nucleotidyl cyclase toxin and ExoY-like effector domains from MARTX toxins. Nat. Commun..

[18] Danchin A. (1993). Phylogeny of adenylyl cyclases. Adv. Second Messenger Phosphoprot. Res..

[19] Gallagher D.T., Kim S.K., Robinson H., Reddy P.T. (2011). Active-site structure of class IV adenylyl cyclase and transphyletic mechanism. J. Mol. Biol..

[20] Gallagher D.T., Smith N.N., Kim S.K., Heroux A., Robinson H., Reddy P.T. (2006). Structure of the class IV adenylyl cyclase reveals a novel fold. J. Mol. Biol..

[21] Raoux-Barbot D., Belyy A., Worpenberg L., Montluc S., Deville C., Henriot V., Velours C., Ladant D., Renault L., Mechold U. (2018). Differential regulation of actin-activated nucleotidyl cyclase virulence factors by filamentous and globular actin. PLoS ONE.

[22] Abeyawardhane D.L., Godoy-Ruiz R., Adipietro K.A., Varney K.M., Rustandi R.R., Pozharski E., Weber D.J. (2021). The Importance of Therapeutically Targeting the Binary Toxin from Clostridioides difficile. Int. J. Mol. Sci..

[23] Smith H., Pinkerton N., Heisler D.B., Kudryashova E., Hall A.R., Karch K.R., Norris A., Wysocki V., Sotomayor M., Reisler E. (2021). Rounding Out the Understanding of ACD Toxicity with the Discovery of Cyclic Forms of Actin Oligomers. Int. J. Mol. Sci..

[24] Juris S.J., Rudolph A.E., Huddler D., Orth K., Dixon J.E. (2000). A distinctive role for the Yersinia protein kinase: Actin binding, kinase activation, and cytoskeleton disruption. Proc. Natl. Acad. Sci. USA.

[25] Kakiuchi S., Yasuda S., Yamazaki R., Teshima Y., Kanda K., Kakiuchi R., Sobue K. (1982). Quantitative determinations of calmodulin in the supernatant and particulate fractions of mammalian tissues. J. Biochem..

[26] Masada N., Ciruela A., Macdougall D.A., Cooper D.M. (2009). Distinct mechanisms of regulation by Ca2+/calmodulin of type 1 and 8 adenylyl cyclases support their different physiological roles. J. Biol. Chem..

[27] Koestler S.A., Rottner K., Lai F., Block J., Vinzenz M., Small J.V. (2009). F- and G-actin concentrations in lamellipodia of moving cells. PLoS ONE.

[28] Singaravelu P., Lee W.L., Wee S., Ghoshdastider U., Ding K., Gunaratne J., Grimes J.M., Swaminathan K., Robinson R.C. (2017). Yersinia effector protein (YopO)-mediated phosphorylation of host gelsolin causes calcium-independent activation leading to disruption of actin dynamics. J. Biol. Chem..

[29] Dessauer C.W., Gilman A.G. (1997). The catalytic mechanism of mammalian adenylyl cyclase. Equilibrium binding and kinetic analysis of P-site inhibition. J. Biol. Chem..

[30] Litvin T.N., Kamenetsky M., Zarifyan A., Buck J., Levin L.R. (2003). Kinetic properties of “soluble” adenylyl cyclase. Synergism between calcium and bicarbonate. J. Biol. Chem..

[31] Guo Q., Shen Y., Zhukovskaya N.L., Florian J., Tang W.J. (2004). Structural and kinetic analyses of the interaction of anthrax adenylyl cyclase toxin with reaction products cAMP and pyrophosphate. J. Biol. Chem..

[32] Stein R.L. (2022). Kinetic Studies of the Activation of Bordetella pertussis Adenylate Cyclase by Calmodulin. Biochemistry.

[33] Steegborn C., Litvin T.N., Levin L.R., Buck J., Wu H. (2005). Bicarbonate activation of adenylyl cyclase via promotion of catalytic active site closure and metal rec.cruitment. Nat. Struct. Mol. Biol..

[34] Gottle M., Dove S., Kees F., Schlossmann J., Geduhn J., Konig B., Shen Y., Tang W.J., Kaever V., Seifert R. (2010). Cytidylyl and uridylyl cyclase activity of bacillus anthracis edema factor and Bordetella pertussis CyaA. Biochemistry.

[35] Beckert U., Wolter S., Hartwig C., Bahre H., Kaever V., Ladant D., Frank D.W., Seifert R. (2014). ExoY from Pseudomonas aeruginosa is a nucleotidyl cyclase with preference for cGMP and cUMP formation. Biochem. Biophys Res. Commun..

[36] Beste K.Y., Spangler C.M., Burhenne H., Koch K.W., Shen Y., Tang W.J., Kaever V., Seifert R. (2013). Nucleotidyl cyclase activity of particulate guanylyl cyclase A: Comparison with particulate guanylyl cyclases E and F, soluble guanylyl cyclase and bacterial adenylyl cyclases CyaA and edema factor. PLoS ONE.

[37] Drum C.L., Yan S.Z., Bard J., Shen Y.Q., Lu D., Soelaiman S., Grabarek Z., Bohm A., Tang W.J. (2002). Structural basis for the activation of anthrax adenylyl cyclase exotoxin by calmodulin. Nature.

[38] Khanppnavar B., Datta S. (2018). Crystal structure and substrate specificity of ExoY, a unique T3SS mediated secreted nucleotidyl cyclase toxin from Pseudomonas aeruginosa. Biochim. Biophys Acta Gen. Subj..

[39] Belyy A., Merino F., Mechold U., Raunser S. (2021). Mechanism of actin-dependent activation of nucleotidyl cyclase toxins from bacterial human pathogens. Nat. Commun..

[40] Arumugham V.B., Ulivieri C., Onnis A., Finetti F., Tonello F., Ladant D., Baldari C.T. (2018). Compartmentalized cyclic AMP production by the Bordetella pertussis and Bacillus anthracis adenylate cyclase toxins differentially affects the immune synapse in T lymphocytes. Front. Immunol..

[41] Zornetta I., Brandi L., Janowiak B., Dal Molin F., Tonello F., Collier R.J., Montecucco C. (2010). Imaging the cell entry of the anthrax oedema and lethal toxins with fluorescent protein chimeras. Cell Microbiol..

[42] Hasan S., Rahman W.U., Sebo P., Osicka R. (2019). Distinct spatiotemporal distribution of bacterial toxin-produced cellular camp differentially inhibits opsonophagocytic signaling. Toxins.

[43] Sayner S.L., Frank D.W., King J., Chen H., VandeWaa J., Stevens T. (2004). Paradoxical cAMP-induced lung endothelial hyperpermeability revealed by pseudomonas aeruginosa ExoY. Circ. Res..

[44] Seifert R., Schirmer B. (2022). cCMP and cUMP come into the spotlight, finally. Trends Biochem. Sci..

[45] Seifert R. (2017). cCMP and cUMP Across the Tree of Life: From cCMP and cUMP Generators to cCMP- and cUMP-Regulated Cell Functions. Handb. Exp. Pharm..

[46] Seifert R., Schneider E.H., Bahre H. (2015). From canonical to non-canonical cyclic nucleotides as second messengers: Pharmacological implications. Pharmacol. Ther..

[47] Weiss A.A., Hewlett E.L., Myers G.A., Falkow S. (1984). Pertussis toxin and extracytoplasmic adenylate cyclase as virulence factors of Bordetella pertussis. J. Infect. Dis..

[48] Chenal A., Ladant D. (2018). Bioengineering of bordetella pertussis adenylate cyclase toxin for antigen-delivery and immunotherapy. Toxins.

[49] Guermonprez P., Khelef N., Blouin E., Rieu P., Ricciardi-Castagnoli P., Guiso N., Ladant D., Leclerc C. (2001). The adenylate cyclase toxin of Bordetella pertussis binds to target cells via the alpha(M)beta(2) integrin (CD11b/CD18). J. Exp. Med..

[50] Eby J.C., Ciesla W.P., Hamman W., Donato G.M., Pickles R.J., Hewlett E.L., Lencer W.I. (2010). Selective translocation of the Bordetella pertussis adenylate cyclase toxin across the basolateral membranes of polarized epithelial cells. J. Biol. Chem..

[51] Donato G.M., Goldsmith C.S., Paddock C.D., Eby J.C., Gray M.C., Hewlett E.L. (2012). Delivery of Bordetella pertussis adenylate cyclase toxin to target cells via outer membrane vesicles. FEBS Lett..

[52] Farfel Z., Friedman E., Hanski E. (1987). The invasive adenylate cyclase of Bordetella pertussis. Intracellular localization and kinetics of penetration into various cells. Biochem. J..

[53] Tang W.J., Krupinski J., Gilman A.G. (1991). Expression and characterization of calmodulin-activated (type I) adenylylcyclase. J. Biol. Chem..

[54] Boyd A.P., Ross P.J., Conroy H., Mahon N., Lavelle E.C., Mills K.H.G. (2005). Bordetella pertussis Adenylate Cyclase Toxin Modulates Innate and Adaptive Immune Responses: Distinct Roles for Acylation and Enzymatic Activity in Immunomodulation and Cell Death. J. Immunol..

[55] Bryn T., Mahic M., Enserink J.M., Schwede F., Aandahl E.M., Taskén K. (2006). The Cyclic AMP-Epac1-Rap1 Pathway Is Dissociated from Regulation of Effector Functions in Monocytes but Acquires Immunoregulatory Function in Mature Macrophages. J. Immunol..

[56] Hall A.B., Gakidis M.A.M., Glogauer M., Wilsbacher J.L., Gao S., Swat W., Brugge J.S. (2006). Requirements for Vav guanine nucleotide exchange factors and Rho GTPases in FcγR- and complement-mediated phagocytosis. Immunity.

[57] Huang Z.-Y., Hunter S., Kim M.-K., Indik Z.K., Schreiber A.D. (2003). The effect of phosphatases SHP-1 and SHIP-1 on signaling by the ITIM- and ITAM-containing Fcγ receptors FcγRIIB and FcγRIIA. J. Leukoc. Biol..

[58] Kamanova J., Kofronova O., Masin J., Genth H., Vojtova J., Linhartova I., Benada O., Just I., Sebo P. (2008). Adenylate Cyclase Toxin Subverts Phagocyte Function by RhoA Inhibition and Unproductive Ruffling. J. Immunol..

[59] Lowell C.A. (2011). Src-family and Syk kinases in activating and inhibitory pathways in innate immune cells: Signaling cross talk. Cold Spring Harb. Perspect. Biol..

[60] Osicka R., Osickova A., Hasan S., Bumba L., Cerny J., Sebo P. (2015). Bordetella adenylate cyclase toxin is a unique ligand of the integrin complement receptor 3. eLife.

[61] Rosales C., Uribe-Querol E. (2017). Phagocytosis: A Fundamental Process in Immunity. BioMed Res. Int..

[62] Cerny O., Anderson K.E., Stephens L.R., Hawkins P.T., Sebo P. (2017). cAMP Signaling of Adenylate Cyclase Toxin Blocks the Oxidative Burst of Neutrophils through Epac-Mediated Inhibition of Phospholipase C Activity. J. Immunol..

[63] Cerny O., Kamanova J., Masin J., Bibova I., Skopova K., Sebo P. (2015). Bordetella pertussis Adenylate Cyclase Toxin Blocks Induction of Bactericidal Nitric Oxide in Macrophages through cAMP-Dependent Activation of the SHP-1 Phosphatase. J. Immunol..

[64] Comer J.E., Chopra A.K., Peterson J.W., König R. (2005). Direct inhibition of T-lymphocyte activation by anthrax toxins in vivo. Infect. Immun..

[65] Dhillon A.S., Pollock C., Steen H., Shaw P.E., Mischak H., Kolch W. (2002). Cyclic AMP-Dependent Kinase Regulates Raf-1 Kinase Mainly by Phosphorylation of Serine 259. Mol. Cell. Biol..

[66] Paccani S.R., Baldari C.T. (2011). T cell targeting by anthrax toxins: Two faces of the same coin. Toxins.

[67] Paccani S.R., Tonello F., Ghittoni R., Natale M., Muraro L., D’Elios M.M., Tang W.J., Montecucco C., Baldari C.T. (2005). Anthrax toxins suppress T lymphocyte activation by disrupting antigen receptor signaling. J. Exp. Med..

[68] Yeager L.A., Chopra A.K., Peterson J.W. (2009). Bacillus anthracis edema toxin suppresses human macrophage phagocytosis and cytoskeletal remodeling via the protein kinase A and exchange protein activated by cyclic AMP pathways. Infect. Immun..

[69] Cleret-Buhot A., Mathieu J., Tournier J.N., Quesnel-Hellmann A. (2012). Both lethal and edema toxins of bacillus anthracis disrupt the human dendritic cell chemokine network. PLoS ONE.

[70] Crawford M.A., Aylott C.V., Bourdeau R.W., Bokoch G.M. (2006). Bacillus anthracis Toxins Inhibit Human Neutrophil NADPH Oxidase Activity. J. Immunol..

[71] Gray M.C., Hewlett E.L. (2011). Cell cycle arrest induced by the bacterial adenylate cyclase toxins from Bacillus anthracis and Bordetella pertussis. Cell. Microbiol..

[72] Ahmad J.N., Cerny O., Linhartova I., Masin J., Osicka R., Sebo P. (2016). cAMP signalling of Bordetella adenylate cyclase toxin through the SHP-1 phosphatase activates the BimEL-Bax pro-apoptotic cascade in phagocytes. Cell. Microbiol..

[73] Paccani S.R., Molin F.D., Benagiano M., Ladant D., D’Elios M.M., Montecucco C., Baldari C.T. (2008). Suppression of T-lymphocyte activation and chemotaxis by the adenylate cyclase toxin of Bordetella pertussis. Infect. Immun..

[74] Dong C., Davis R.J., Flavell R.A. (2002). MAP kinases in the immune response. Annu. Rev. Immunol..

[75] Viola A., Contento R.L., Molon B. (2006). T cells and their partners: The chemokine dating agency. Trends Immunol..

[76] Howe A.K. (2004). Regulation of actin-based cell migration by cAMP/PKA. Biochim. Biophys. Acta Mol. Cell Res..

[77] Angely C., Ladant D., Planus E., Louis B., Filoche M., Chenal A., Isabey D. (2020). Functional and structural consequences of epithelial cell invasion by.y Bordetella pertussis adenylate cyclase toxin. PLoS ONE.

[78] Hasan S., Kulkarni N.N., Asbjarnarson A., Linhartova I., Osicka R., Sebo P., Gudmundsson G.H. (2018). Bordetella pertussis adenylate cyclase toxin disrupts functional integrity of bronchial epithelial layers. Infect. Immun..

[79] Bradley K.A., Mogridge J., Mourez M., Collier R.J., Young J.A.T. (2001). Identification of the cellular receptor for anthrax toxin. Nature.

[80] Scobie H.M., Rainey G.J.A., Bradley K.A., Young J.A.T. (2003). Human capillary morphogenesis protein 2 functions as an anthrax toxin receptor. Proc. Natl. Acad. Sci. USA.

[81] Young J.A., Collier R.J. (2007). Anthrax toxin: Receptor binding, internalization, pore formation, and translocation. Annu. Rev. Biochem..

[82] Blaustein R.O., Koehler T.M., Collier R.J., Finkelstein A. (1989). Anthrax toxin: Channel-forming activity of protective antigen in planar phospholipid bilayers. Proc. Natl. Acad. Sci. USA.

[83] Paccani S.R., Tonello F., Patrussi L., Capitani N., Simonato M., Montecucco C., Baldari C.T. (2007). Anthrax toxins inhibit immune cell chemotaxis by perturbing chemokine receptor signalling. Cell. Microbiol..

[84] Gnade B.T., Moen S.T., Chopra A.K., Peterson J.W., Yeager L.A. (2010). Emergence of anthrax edema toxin as a master manipulator of macrophage and B cell functions. Toxins.

[85] Guichard A., McGillivray S.M., Cruz-Moreno B., Van Sorge N.M., Nizet V., Bier E. (2010). Anthrax toxins cooperatively inhibit endocytic recycling by the Rab11/Sec15 exocyst. Nature.

[86] Guichard A., Jain P., Moayeri M., Schwartz R., Chin S., Zhu L., Cruz-Moreno B., Liu J.Z., Aguilar B., Hollands A. (2017). Anthrax edema toxin disrupts distinct steps in Rab11-dependent junctional transport. PLoS Pathog..

[87] Maddugoda M.P., Stefani C., Gonzalez-Rodriguez D., Saarikangas J., Torrino S., Janel S., Munro P., Doye A., Prodon F., Aurrand-Lions M. (2011). CAMP signaling by Anthrax edema toxin induces transendothelial cell tunnels, which Are resealed by MIM via Arp2/3-Driven actin polymerization. Cell Host Microbe.

[88] Carman C.V., Springer T.A. (2008). Trans-cellular migration: Cell-cell contacts get intimate. Curr. Opin. Cell Biol..

[89] Stefani C., Gonzalez-Rodriguez D., Senju Y., Doye A., Efimova N., Janel S., Lipuma J., Tsai M.C., Hamaoui D., Maddugoda M.P. (2017). Ezrin enhances line tension along transcellular tunnel edges via NMIIa driven actomyosin cable formation. Nat. Commun..

[90] Boyer L., Doye A., Rolando M., Flatau G., Munro P., Gounon P., Clément R., Pulcini C., Popoff M.R., Mettouchi A. (2006). Induction of transient macroapertures in endothelial cells through RhoA inhibition by Staphylococcus aureus factors. J. Cell Biol..

[91] Rolando M., Munro P., Stefani C., Auberger P., Flatau G., Lemichez E. (2009). Injection of Staphylococcus aureus EDIN by the Bacillus anthracis protective antigen machinery induces vascular permeability. Infect. Immun..

[92] Hong J., Beeler J., Zhukovskaya N.L., He W., Tang W.J., Rosner M.R. (2005). Anthrax edema factor potency depends on mode of cell entry. Biochem. Biophys. Res. Commun..

[93] Kloth C., Schirmer B., Munder A., Stelzer T., Rothschuh J., Seifert R. (2018). The Role of Pseudomonas aeruginosa ExoY in an Acute Mouse Lung Infection Mo.odel. Toxins.

[94] Belyy A., Santecchia I., Renault L., Bourigault B., Ladant D., Mechold U. (2018). The extreme C terminus of the Pseudomonas aeruginosa effector ExoY is crucial for binding to its eukaryotic activator, F-actin. J. Biol. Chem..

[95] Silistre H., Raoux-Barbot D., Mancinelli F., Sangouard F., Dupin A., Belyy A., Deruelle V., Renault L., Ladant D., Touqui L. (2021). Prevalence of ExoY Activity in Pseudomonas aeruginosa Reference Panel Strains and Impact on Cytotoxicity in Epithelial Cells. Front. Microbiol..

[96] Huber P., Bouillot S., Elsen S., Attrée I. (2014). Sequential inactivation of Rho GTPases and Lim kinase by Pseudomonas aeruginosa toxins ExoS and ExoT leads to endothelial monolayer breakdown. Cell. Mol. Life Sci..

[97] Schulert G.S., Feltman H., Rabin S.D., Martin C.G., Battle S.E., Rello J., Hauser A.R. (2003). Secretion of the toxin ExoU is a marker for highly virulent Pseudomonas aeruginosa isolates obtained from patients with hospital-acquired pneumonia. J. Infect. Dis..

[98] Woida P.J., Satchell K.J.F. (2020). The Vibrio cholerae MARTX toxin silences the inflammatory response to cytoskeletal damage before inducing actin cytoskeleton collapse. Sci. Signal..

[99] Mancl J.M., Suarez C., Liang W.G., Kovar D.R., Tang W.J. (2020). Pseudomonas aeruginosa exoenzyme Y directly bundles actin filaments. J. Biol. Chem..

[100] Kudryashova E., Heisler D.B., Kudryashov D.S. (2017). Pathogenic Mechanisms of Actin Cross-Linking Toxins: Peeling Away the Layers. Curr. Top. Microbiol. Immunol..

[101] Teixeira-Nunes M., Retailleau P., Raoux-Barbot D., Plancqueel S., Missinou A.A., Comisso M., Legrand P., Velours C., Ladant D., Mechold U. Structural basis for the activation of bacterial ExoY nucleotidyl cyclase exotoxins by free or profilin-bound G-actin and impact of the ternary complexes on actin polymerization in vitro. submitted.

[102] Sievers F., Wilm A., Dineen D., Gibson T.J., Karplus K., Li W., Lopez R., McWilliam H., Remmert M., Soding J. (2011). Fast, scalable generation of high-quality protein multiple sequence alignments using Clustal Omega. Mol. Syst. Biol..

[103] Dereeper A., Guignon V., Blanc G., Audic S., Buffet S., Chevenet F., Dufayard J.F., Guindon S., Lefort V., Lescot M. (2008). Phylogeny.fr: Robust phylogenetic analysis for the non-specialist. Nucleic Acids Res..

[104] Jeon J., Kim Y.J., Shin H., Ha U.H. (2017). T3SS effector ExoY reduces inflammasome-related responses by suppressing bacterial motility and delaying activation of NF-kappaB and caspase-1. FEBS J..

[105] Liu T., Zhang L., Joo D., Sun S.C. (2017). NF-κB signaling in inflammation. Signal. Transduct. Target. Ther..

[106] Bergsbaken T., Fink S.L., Cookson B.T. (2009). Pyroptosis: Host cell death. Nat. Rev. Microbiol..

[107] He C., Zhou Y., Liu F., Liu H., Tan H., Jin S., Wu W., Ge B. (2017). Bacterial nucleotidyl cyclase inhibits the host innate immune response by suppressing TAK1 activation. Infect. Immun..

[108] Shi J.H., Sun S.C. (2018). Tumor necrosis factor receptor-associated factor regulation of nuclear factor κB and mitogen-activated protein kinase pathways. Front. Immunol..

[109] Balczon R., Prasain N., Ochoa C., Prater J., Zhu B., Alexeyev M., Sayner S., Frank D.W., Stevens T. (2013). Pseudomonas aeruginosa Exotoxin Y-Mediated Tau Hyperphosphorylation Impairs Microtubule Assembly in Pulmonary Microvascular Endothelial Cells. PLoS ONE.

[110] Ochoa C.D., Alexeyev M., Pastukh V., Balczon R., Stevens T. (2012). Pseudomonas aeruginosa exotoxin Y is a promiscuous cyclase that increases endothelial tau phosphorylation and permeability. J. Biol. Chem..

[111] Prasain N., Alexeyev M., Balczon R., Stevens T. (2009). Soluble adenylyl cyclase-dependent microtubule disassembly reveals a novel mechanism of endothelial cell retraction. Am. J. Physiol. Lung Cell. Mol. Physiol..

[112] Hritonenko V., Mun J.J., Tam C., Simon N.C., Barbieri J.T., Evans D.J., Fleiszig S.M.J. (2011). Adenylate cyclase activity of Pseudomonas aeruginosa ExoY can mediate bleb-niche formation in epithelial cells and contributes to virulence. Microb. Pathog..

[113] Cowell B.A., Evans D.J., Fleiszig S.M.J. (2005). Actin cytoskeleton disruption by ExoY and its effects on Pseudomonas aeruginosa invasion. FEMS Microbiol. Lett..

[114] Verin A.D., Birukova A., Wang P., Feng L., Becker P., Birukov K., Garcia J.G.N. (2001). Microtubule disassembly increases endothelial cell barrier dysfunction: Role of MLC phosphorylation. Am. J. Physiol. Lung Cell. Mol. Physiol..

[115] Stevens T.C., Ochoa C.D., Morrow K.A., Robson M.J., Prasain N., Zhou C., Alvarez D.F., Frank D.W., Balczon R., Stevens T. (2014). The Pseudomonas aeruginosa exoenzyme Y impairs endothelial cell proliferation and vascular repair following lung injury. Am. J. Physiol. Lung Cell Mol. Physiol..

[116] Voth S., Gwin M., Francis C.M., Balczon R., Frank D.W., Pittet J.F., Wagener B.M., Moser S.A., Alexeyev M., Housley N. (2020). Virulent Pseudomonas aeruginosa infection converts antimicrobial amyloids into cytotoxic prions. FASEB J..

[117] Renema P., Kozhukhar N., Pastukh V., Spadafora D., Paudel S.S., Tambe D.T., Alexeyev M., Frank D.W., Stevens T. (2020). Exoenzyme Y induces extracellular active caspase-7 accumulation independent from apoptosis: Modulation of transmissible cytotoxicity. Am. J. Physiol. Lung Cell Mol. Physiol..

[118] Welch M.D., Way M. (2013). Arp2/3-mediated actin-based motility: A tail of pathogen abuse. Cell Host Microbe.

[119] Purde V., Busch F., Kudryashova E., Wysocki V.H., Kudryashov D.S. (2019). Oligomerization Affects the Ability of Human Cyclase-Associated Proteins 1 and 2 to Promote Actin Severing by Cofilins. Int. J. Mol. Sci..

[120] Van Nhieu G.T., Romero S. (2017). Common Themes in Cytoskeletal Remodeling by Intracellular Bacterial Effectors. Handb. Exp. Pharm..

[121] Aszodi A., Pfeifer A., Ahmad M., Glauner M., Zhou X.H., Ny L., Andersson K.E., Kehrel B., Offermanns S., Fassler R. (1999). The vasodilator-stimulated phosphoprotein (VASP) is involved in cGMP- and cAMP-mediated inhibition of agonist-induced platelet aggregation, but is dispensable for smooth muscle function. EMBO J..

[122] Gertler F.B., Niebuhr K., Reinhard M., Wehland J., Soriano P. (1996). Mena, a relative of VASP and Drosophila Enabled, is implicated in the control of microfilament dynamics. Cell.

[123] Halbrugge M., Friedrich C., Eigenthaler M., Schanzenbacher P., Walter U. (1990). Stoichiometric and reversible phosphorylation of a 46-kDa protein in human platelets in response to cGMP- and cAMP-elevating vasodilators. J. Biol. Chem..

[124] Simao M., Regnier F., Taheraly S., Fraisse A., Tacine R., Fraudeau M., Benabid A., Feuillet V., Lambert M., Delon J. (2021). cAMP Bursts Control T Cell Directionality by Actomyosin Cytoskeleton Remodeling. Front. Cell Dev. Biol..

[125] Sehrawat S., Cullere X., Patel S., Italiano J., Mayadas T.N. (2008). Role of Epac1, an exchange factor for Rap GTPases, in endothelial microtubule dynamics and barrier function. Mol. Biol. Cell.

[126] Kooistra M.R., Dube N., Bos J.L. (2007). Rap1: A key regulator in cell-cell junction formation. J. Cell Sci..

[127] Kazmierczak B.I., Engel J.N. (2002). Pseudomonas aeruginosa ExoT acts in vivo as a GTPase-activating protein for RhoA, Rac1, and Cdc42. Infect. Immun..

[128] Pederson K.J., Vallis A.J., Aktories K., Frank D.W., Barbieri J.T. (1999). The amino-terminal domain of Pseudomonas aeruginosa ExoS disrupts actin filaments via small-molecular-weight GTP-binding proteins. Mol. Microbiol..

[129] Maresso A.W., Baldwin M.R., Barbieri J.T. (2004). Ezrin/radixin/moesin proteins are high affinity targets for ADP-ribosylation by Pseudomonas aeruginosa ExoS. J. Biol. Chem..

[130] Deng Q., Sun J., Barbieri J.T. (2005). Uncoupling Crk signal transduction by Pseudomonas exoenzyme T. J. Biol. Chem..

[131] Sun J., Barbieri J.T. (2003). Pseudomonas aeruginosa ExoT ADP-ribosylates CT10 regulator of kinase (Crk) proteins. J. Biol. Chem..

[132] Wood S., Goldufsky J., Shafikhani S.H. (2015). Pseudomonas aeruginosa ExoT Induces Atypical Anoikis Apoptosis in Target Host Cells by Transforming Crk Adaptor Protein into a Cytotoxin. PLoS Pathog..

[133] Ichikawa J.K., English S.B., Wolfgang M.C., Jackson R., Butte A.J., Lory S. (2005). Genome-wide analysis of host responses to the Pseudomonas aeruginosa type III secretion system yields synergistic effects. Cell. Microbiol..

[134] Zhou Y., Huang C., Yin L., Wan M., Wang X., Li L., Liu Y., Wang Z., Fu P., Zhang N. (2017). Nϵ-Fatty acylation of Rho GTPases by a MARTX toxin effector. Science.

[135] Woida P.J., Satchell K.J.F. (2018). Coordinated delivery and function of bacterial MARTX toxin effectors. Mol. Microbiol..

[136] Tesmer J.J., Sunahara R.K., Gilman A.G., Sprang S.R. (1997). Crystal structure of the catalytic domains of adenylyl cyclase in a complex with Gsalpha.GTPgammaS. Science.

[137] Guo Q., Shen Y., Lee Y.S., Gibbs C.S., Mrksich M., Tang W.J. (2005). Structural basis for the interaction of Bordetella pertussis adenylyl cyclase toxin with calmodulin. EMBO J..

[138] Kleinboelting S., Diaz A., Moniot S., van den Heuvel J., Weyand M., Levin L.R., Buck J., Steegborn C. (2014). Crystal structures of human soluble adenylyl cyclase reveal mechanisms of catalysis and of its activation through bicarbonate. Proc. Natl. Acad. Sci. USA.

[139] Linder J.U. (2006). Class III adenylyl cyclases: Molecular mechanisms of catalysis and regulation. Cell Mol. Life Sci..

[140] Dove S. (2017). Mammalian Nucleotidyl Cyclases and Their Nucleotide Binding Sites. Handb. Exp. Pharm..

[141] Childers K.C., Garcin E.D. (2018). Structure/function of the soluble guanylyl cyclase catalytic domain. Nitric Oxide.

[142] Yarwood S.J. (2020). Special Issue on “New Advances in Cyclic AMP Signalling”-An Editorial Overview. Cells.

[143] Tesmer J.J., Sunahara R.K., Johnson R.A., Gosselin G., Gilman A.G., Sprang S.R. (1999). Two-metal-Ion catalysis in adenylyl cyclase. Science.

[144] Kamenetsky M., Middelhaufe S., Bank E.M., Levin L.R., Buck J., Steegborn C. (2006). Molecular details of cAMP generation in mammalian cells: A tale of two systems. J. Mol. Biol..

[145] Munier H., Bouhss A., Krin E., Danchin A., Gilles A.M., Glaser P., Barzu O. (1992). The role of histidine 63 in the catalytic mechanism of Bordetella pertussis adenylate cyclase. J. Biol. Chem..

[146] Glaser P., Elmaoglou-Lazaridou A., Krin E., Ladant D., Barzu O., Danchin A. (1989). Identification of residues essential for catalysis and binding of calmodulin in Bordetella pertussis adenylate cyclase by site-directed mutagenesis. EMBO J..

[147] Gupta M., Alam S., Bhatnagar R. (2006). Kinetic characterization and ligand binding studies of His351 mutants of Bacillus anthracis adenylate cyclase. Arch. Biochem. Biophys..

[148] Hahn D.K., Tusell J.R., Sprang S.R., Chu X. (2015). Catalytic Mechanism of Mammalian Adenylyl Cyclase: A Computational Investigation. Biochemistry.

[149] Mones L., Tang W.J., Florian J. (2013). Empirical valence bond simulations of the chemical mechanism of ATP to cAMP conversion by anthrax edema factor. Biochemistry.

[150] Jara G.E., Martinez L. (2016). Anthrax Edema Factor: An Ion-Adaptive Mechanism of Catalysis with Increased Transition-State Conformational Flexibility. J. Phys. Chem. B.

[151] Dessauer C.W., Tesmer J.J., Sprang S.R., Gilman A.G. (1999). The interactions of adenylate cyclases with P-site inhibitors. Trends Pharm. Sci..

[152] Adasme M.F., Linnemann K.L., Bolz S.N., Kaiser F., Salentin S., Haupt V.J., Schroeder M. (2021). PLIP 2021: Expanding the scope of the protein-ligand interaction profiler to DNA and RNA. Nucleic Acids Res..

[153] Steegborn C. (2014). Structure, mechanism, and regulation of soluble adenylyl cyclases - similarities and differences to transmembrane adenylyl cyclases. Biochim. Biophys. Acta.

[154] Lubker C., Seifert R. (2015). Effects of 39 Compounds on Calmodulin-Regulated Adenylyl Cyclases AC1 and Bacillus anthracis Edema Factor. PLoS ONE.

[155] Londos C., Wolff J. (1977). Two distinct adenosine-sensitive sites on adenylate cyclase. Proc. Natl. Acad. Sci. USA.

[156] Johnson R.A., Shoshani I. (1990). Inhibition of Bordetella pertussis and Bacillus anthracis adenylyl cyclases by polyadenylate and “P”-site agonists. J. Biol. Chem..

[157] Shen Y., Zhukovskaya N.L., Zimmer M.I., Soelaiman S., Bergson P., Wang C.R., Gibbs C.S., Tang W.J. (2004). Selective inhibition of anthrax edema factor by adefovir, a drug for chronic hepatitis B virus infection. Proc. Natl. Acad. Sci. USA.

[158] Soelaiman S., Wei B.Q., Bergson P., Lee Y.S., Shen Y., Mrksich M., Shoichet B.K., Tang W.J. (2003). Structure-based inhibitor discovery against adenylyl cyclase toxins from pathogenic bacteria that cause anthrax and whooping cough. J. Biol. Chem..

[159] Lee Y.S., Bergson P., He W.S., Mrksich M., Tang W.J. (2004). Discovery of a small molecule that inhibits the interaction of anthrax edema factor with its cellular activator, calmodulin. Chem. Biol..

[160] Laine E., Goncalves C., Karst J.C., Lesnard A., Rault S., Tang W.J., Malliavin T.E., Ladant D., Blondel A. (2010). Use of allostery to identify inhibitors of calmodulin-induced activation of Bacillus anthracis edema factor. Proc. Natl. Acad. Sci. USA.

[161] Seifert R., Dove S. (2013). Inhibitors of Bacillus anthracis edema factor. Pharmacol. Ther..

[162] Hauser A.R., Cobb E., Bodi M., Mariscal D., Valles J., Engel J.N., Rello J. (2002). Type III protein secretion is associated with poor clinical outcomes in patients with ventilator-associated pneumonia caused by Pseudomonas aeruginosa. Crit. Care Med..

[163] Wiersinga W.J., van der Poll T., White N.J., Day N.P., Peacock S.J. (2006). Melioidosis: Insights into the pathogenicity of Burkholderia pseudomallei. Nat. Rev. Microbiol..

[164] Tal N., Morehouse B.R., Millman A., Stokar-Avihail A., Avraham C., Fedorenko T., Yirmiya E., Herbst E., Brandis A., Mehlman T. (2021). Cyclic CMP and cyclic UMP mediate bacterial immunity against phages. Cell.

